# Characterization of a Four-Component Regulatory System Controlling Bacteriocin Production in Streptococcus gallolyticus

**DOI:** 10.1128/mBio.03187-20

**Published:** 2021-01-05

**Authors:** Alexis Proutière, Laurence du Merle, Bruno Périchon, Hugo Varet, Myriam Gominet, Patrick Trieu-Cuot, Shaynoor Dramsi

**Affiliations:** a Unité de Biologie des Bactéries Pathogènes à Gram-positif, Institut Pasteur, Paris, France; b CNRS Unité Mixte de Recherches, UMR 2001, Paris, France; c Université de Paris, 75013, Paris, France; d Institut Pasteur, Hub Bioinformatique et Biostatistique, Département de Biologie Computationnelle (USR 3756 IP CNRS), Paris, France; e Institut Pasteur, Biomics Platform, Centre de Ressources et Recherches Technologiques, Paris, France; University of Illinois at Chicago; University of Minnesota Medical School

**Keywords:** *Streptococcus*, bacteriocins, regulation of gene expression

## Abstract

Bacteriocins are natural antimicrobial peptides produced by bacteria to kill closely related competitors. The opportunistic pathogen Streptococcus gallolyticus subsp. *gallolyticus* was recently shown to outcompete commensal enterococci of the murine microbiota under tumoral conditions thanks to the production of a two-peptide bacteriocin named gallocin. Here, we identified four genes involved in the regulatory control of gallocin in S. gallolyticus subsp. *gallolyticus* UCN34 that encode a histidine kinase/response regulator two-component system (BlpH/BlpR), a secreted peptide (GSP [gallocin-stimulating peptide]), and a putative regulator of unknown function (BlpS). While BlpR is a typical 243-amino-acid (aa) response regulator possessing a phospho-receiver domain and a LytTR DNA-binding domain, BlpS is a 108-aa protein containing only a LytTR domain. Our results showed that the secreted peptide GSP activates the dedicated two-component system BlpH/BlpR to induce gallocin transcription. A genome-wide transcriptome analysis indicates that this regulatory system (GSP-BlpH/BlpR) is specific for bacteriocin production. Importantly, as opposed to BlpR, BlpS was shown to repress gallocin gene transcription. A conserved operator DNA sequence of 30 bp was found in all promoter regions regulated by BlpR and BlpS. Electrophoretic mobility shift assays (EMSA) and footprint assays showed direct and specific binding of BlpS and BlpR to various regulated promoter regions in a dose-dependent manner on this conserved sequence. Gallocin expression appears to be tightly controlled in S. gallolyticus subsp. *gallolyticus* by quorum sensing and antagonistic activity of 2 LytTR-containing proteins. Competition experiments in gut microbiota medium and 5% CO_2_ to mimic intestinal conditions demonstrate that gallocin is functional under these *in vivo*-like conditions.

## INTRODUCTION

Streptococcus gallolyticus subsp. *gallolyticus*, formerly known as Streptococcus bovis biotype I, is an opportunistic Gram-positive pathogen responsible for septicemia and endocarditis in the elderly (1). Invasive S. gallolyticus subsp. *gallolyticus* infections are strongly associated with asymptomatic colonic neoplasia, but the mechanisms underlying this association are still unclear (2, 3). Recently, it was shown that S. gallolyticus subsp. *gallolyticus* produces a specific bacteriocin named gallocin, whose antimicrobial activity is enhanced by the increased level of secondary bile salts observed under tumoral conditions, allowing S. gallolyticus subsp. *gallolyticus* to colonize the murine gut by killing resident enterococci (4). As such, gallocin constitutes the first bacterial factor which could explain the S. gallolyticus subsp. *gallolyticus* association with colonic tumors.

Bacteriocins are natural antimicrobial peptides produced by many bacteria. Producer strains are protected from their own bacteriocins by the presence of an immunity system. Most bacteriocins have a narrow spectrum of activity restricted to bacteria closely related to the producer. Therefore, bacteriocin production is important for the colonization of specific niches, especially in competitive environments such as the gut (5). Bacteriocins of Gram-positive bacteria have been divided into four classes based on size, amino acid composition, and structure (6). Class I includes small (<5-kDa) linear peptides containing posttranslationally modified amino acids called lantibiotics, class II includes small (<10-kDa) linear peptides without posttranslationally modified amino acids, class III includes large (>10-kDa) proteins, and class IV includes small cyclic peptides. Class II bacteriocins are further subdivided into three groups: class IIa consists of pediocin-like bacteriocins, class IIb consists of bacteriocins with two or more peptides, and class IIc consists of all other bacteriocins not fitting in classes IIa and IIb. *In silico* analysis indicates that gallocin likely belongs to class IIb bacteriocins (4). In general, these bacteriocins kill susceptible strains by forming pores in the target membranes, resulting in ion leakage and cell death (7).

Some class IIb bacteriocin loci encode a three-component regulatory system composed of an inducing peptide and a dedicated two-component system (TCS) with a membrane-bound histidine kinase and a cytoplasmic response regulator. Activation of bacteriocin production through this regulatory system is similar to quorum sensing regulatory systems. First, the inducing peptide is secreted into the extracellular medium and, upon reaching a threshold concentration, binds to and activates the histidine kinase, resulting in phosphorylation of its associated response regulator. The phosphorylated response regulator then activates the transcription of genes necessary for class IIb bacteriocin production, including its own transcription, resulting in a rapid overexpression of the regulated genes (7–9). In streptococci, complex regulatory cross talk has been identified between bacteriocin production and competence (10). Natural competence has been reported in the S. bovis group (11) but not in S. gallolyticus subsp. *gallolyticus*. A peptide previously identified in the extracellular medium of S. gallolyticus subsp. *gallolyticus* called CSP (due to its similarity to competence-stimulating peptide) was shown to induce bacteriocin production but did not allow capture and integration of foreign plasmid DNA (12). In this report and in the accompanying paper (13), we propose that CSP should be renamed GSP, for “gallocin-stimulating peptide.”

The aim of the present study was to identify and characterize the regulatory system involved in gallocin production and to identify other potentially coregulated genes. In addition to the typical three-component system, consisting of an inducing peptide (GSP), a dedicated histidine kinase (BlpH), and a response regulator (BlpR), a fourth regulatory component named BlpS, which inhibits gallocin expression, was identified in this work. Combining genetics and biochemical analyses, we propose a model describing the tight regulation of gallocin expression through GSP/BlpHR/BlpS. Moreover, the presence of several putative novel bacteriocins coexpressed with gallocin highlights the importance of these antimicrobial peptides for the gut colonization by this pathobiont associated with colorectal cancer.

## RESULTS

### Identification of a dedicated three-component regulatory system involved in gallocin production.

**FIG 1:**
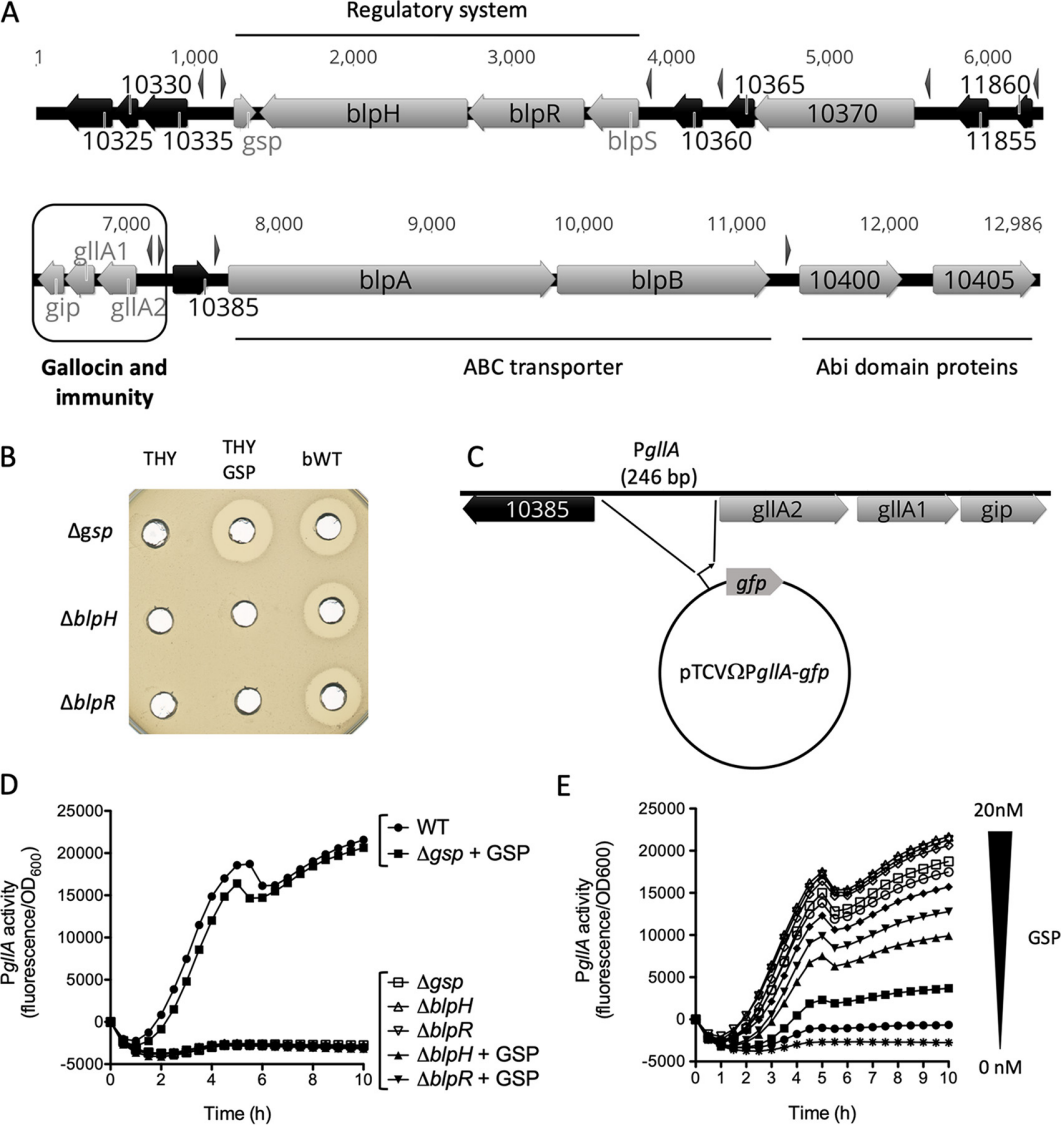
A three-component system activates gallocin transcription in S. gallolyticus subsp. *gallolyticus* UCN34. (A) Gallocin locus in strain UCN34 (12,986 bp) extending from *gallo_rs10325* to *gallo_rs10405* (UCN34 genome reference NC_013798.1; new annotation). Genes with a predicted function are in gray; hypothetical genes are in black. Gene names are those given in this work or referred to using the novel “*gallo_rs*” annotation (e.g., “10325” for *gallo_rs10325*). Arrowheads above the genes indicate the presence of a 30-bp conserved motif in the promoter regions. (B) Agar diffusion assay revealing the capacity of S. gallolyticus subsp. *gallolyticus* UCN34 Δ*gsp*, Δ*blpH*, and Δ*blpR* to inhibit the growth of the gallocin-sensitive S. gallolyticus subsp. *macedonicus* strain. Mutants were cultured either in THY or in THY supplemented with 20 nM synthetic GSP (THY GSP). Activity of one counterpart that reverted back to WT (bWT) is also shown on the right. (C) Schematic representation of the reporter plasmid pTCVΩP*gllA*-*gfp* to monitor gallocin promoter activity. (D) P*gllA* activity (fluorescence divided by OD_600_) in strain UCN34 WT, Δ*gsp*, Δ*blpH*, and Δ*blpR* in presence or absence of 20 nM synthetic GSP. One representative curve of three independent experiments is shown here for each condition. (E) P*gllA* activity in S. gallolyticus subsp. *gallolyticus* UCN34 Δ*gsp* containing the reporter plasmid in the presence of growing concentrations of synthetic GSP (curves from bottom to top were obtained in culture medium containing 0 to 20 nM GSP, respectively; the concentration increasing by 2 nM between each curve. The three upper curves were obtained with 16, 18, and 20 nM GSP. One representative curve of three independent experiments is shown for each condition.

To understand how gallocin production is regulated in S. gallolyticus subsp. *gallolyticus* UCN34 (14), the genomic locus encoding this putative class IIb bacteriocin was inspected for the presence of potential regulatory genes. Genes encoding a three-component regulatory system were identified at one end of the gallocin locus (Fig. 1A). This module is composed of 3 genes: *blpH*, encoding a putative histidine kinase, *blpR*, encoding a putative response regulator, and a divergently transcribed gene encoding a putative inducing peptide named GSP (gallocin-stimulating peptide). The regulatory genes are close to the genes encoding the gallocin peptides, recently renamed *gllA1* and *gllA2* (15), the gene encoding the putative immunity peptide (*gip* for gallocin immunity peptide), two genes encoding an ABC transporter (*blpA* and *blpB*) shown to be involved in gallocin peptide secretion (13), and genes for other conserved bacteriocin-associated proteins, such as Abi domain proteins (*gallo_rs10400* and *gallo_rs10405*) (Fig. 1A).

A genetic approach was undertaken to demonstrate the role of these three regulatory genes in gallocin production. Markerless in-frame deletion mutants of *gsp*, *blpH*, and *blpR* were obtained in S. gallolyticus subsp. *gallolyticus* UCN34. For each mutant, we also selected a clone that reverted to the wild-type genotype (bWT) following homologous recombination. Gallocin production is easily visualized through its antimicrobial activity against the very closely related bacterium Streptococcus gallolyticus subsp. *macedonicus* (Fig. S1A), which was used as a susceptible indicator strain throughout this work. As shown in Fig. 1B, gallocin production was abolished in the Δ*gsp*, Δ*blpH*, and Δ*blpR* mutants compared to their bWT strains. All three mutants did not exhibit any killing activity against the S. gallolyticus subsp. *macedonicus* prey strain, indicating that these three genes are essential for gallocin production in S. gallolyticus subsp. *gallolyticus* UCN34. We reasoned that if *gsp* encodes a secreted inducing peptide that activates its cognate two-component system, addition of GSP peptide to the extracellular medium should restore gallocin production by the Δ*gsp* mutant. We also hypothesized that GSP, like other inducing peptides, is synthesized as a precursor matured by cleavage upon secretion after a double glycine motif (16). The predicted mature GSP peptide corresponding to the 24 C-terminal amino acids encoded by *gsp* was synthesized chemically (Fig. S1B). Addition of synthetic GSP to the culture medium restored gallocin production by the Δ*gsp* mutant (Fig. 1B). Importantly, addition of GSP did not restored gallocin production in the Δ*blpH* or Δ*blpR* mutants, suggesting that GSP activates transcription of genes involved in gallocin production through the BlpHR TCS.

10.1128/mBio.03187-20.1 FIG S1 Susceptibility of S. gallolyticus subsp. *macedonicus* to gallocin and GSP amino acid sequence. (A) Agar diffusion assay of S. gallolyticus subsp. *gallolyticus* UCN34 WT and Δ*blp* supernatant on a lawn of S. gallolyticus subsp. *macedonicus*. S. gallolyticus subsp. *gallolyticus* Δ*blp* (4) has the whole gallocin operon (*gllA1*-*gallA2*-*gip*) deleted. (B) Amino acid sequence of GSP obtained by translation of *gsp* gene. The predicted cleavage site, indicated by a black arrowhead, occurs after the double glycine motif in red. The synthetic peptide used in this study is composed of the 24 C-terminal residues in bold. The white arrowhead indicates the cleavage site identified after GSP purification from culture supernatant and sequencing (12, 13). Download FIG S1, TIF file, 0.1 MB. Copyright © 2021 Proutière et al. 2021 Proutière et al. This content is distributed under the terms of the Creative Commons Attribution 4.0 International license.

To demonstrate that this regulatory system activates gallocin gene transcription, we constructed a reporter plasmid expressing *gfp* under the control of the gallocin operon promoter (pTCVΩP*gllA*-*gfp*) to monitor the promoter activity by recording green fluorescent protein (GFP) fluorescence during growth (Fig. 1C). As shown in Fig. S2, P*gllA* activity in S. gallolyticus subsp. *gallolyticus* UCN34 WT was null at the beginning of the culture, increased throughout growth, and was maximal at the end of the exponential phase. The persistence of the GFP signal beyond the late log phase was observed in the M9Y medium but not consistently in THY. To more thoroughly investigate gallocin gene expression along the growth curve, quantitative reverse transcriptase PCR (qRT-PCR) experiments on selected genes were performed at various time points (early exponential, exponential, late exponential, and stationary phases). All the genes examined except *gsp* displayed a characteristic bell curve shape, with maximal expression during late exponential phase. In contrast, *gsp* expression was maximal much earlier in growth, during exponential phase (data not shown).

10.1128/mBio.03187-20.2 FIG S2 Monitoring GFP fluorescence using P*gllA*- or P*tetO*-inducible promoters. P*gllA* is active in S. gallolyticus subsp. *gallolyticus* UCN34 WT but not in GBS NEM316. P*tetO* was induced with 200 ng/ml of anhydrotetracycline (AnTc) in S. gallolyticus subsp. *gallolyticus* UCN34. All the strains exhibited similar growth. For clarity, only the growth curve of S. gallolyticus subsp. *gallolyticus* WT (pTCVΩ*PgllA*-*gfp)* is presented as a dotted curve. One representative curve of three independent experiments is shown for each condition. Download FIG S2, TIF file, 0.1 MB. Copyright © 2021 Proutière et al. 2021 Proutière et al. This content is distributed under the terms of the Creative Commons Attribution 4.0 International license.

Next, we showed that P*gllA* was completely inactive in Streptococcus agalactiae NEM316, which does not contain the specific regulatory system *gsp*-*blpRH* (Fig. S2). This result demonstrates that gallocin promoter activity depends on a S. gallolyticus subsp. *gallolyticus*-specific regulatory system. Consistently, the P*gllA* promoter was totally inactive in the three regulatory Δ*gsp*, Δ*blpH*, *and* Δ*blpR* mutants (Fig. 1D). Addition of increasing concentrations of GSP (2 to 20 nM) to the culture medium restored gallocin promoter activity in a dose-dependent manner in the S. gallolyticus subsp. *gallolyticus* Δ*gsp* mutant but not in the Δ*blpH* and Δ*blpR* mutants (Fig. 1D and E), confirming that GSP activates gallocin gene transcription through the BlpRH TCS. As shown in Fig. 1E, GSP is active at very low concentrations (6 nM), and maximal activation of gallocin promoter was recorded with 16 nM GSP.

### Identification of the regulon controlled by the three-component regulatory system GSP/BlpHR in S. gallolyticus subsp. *gallolyticus*.

**FIG 2:**
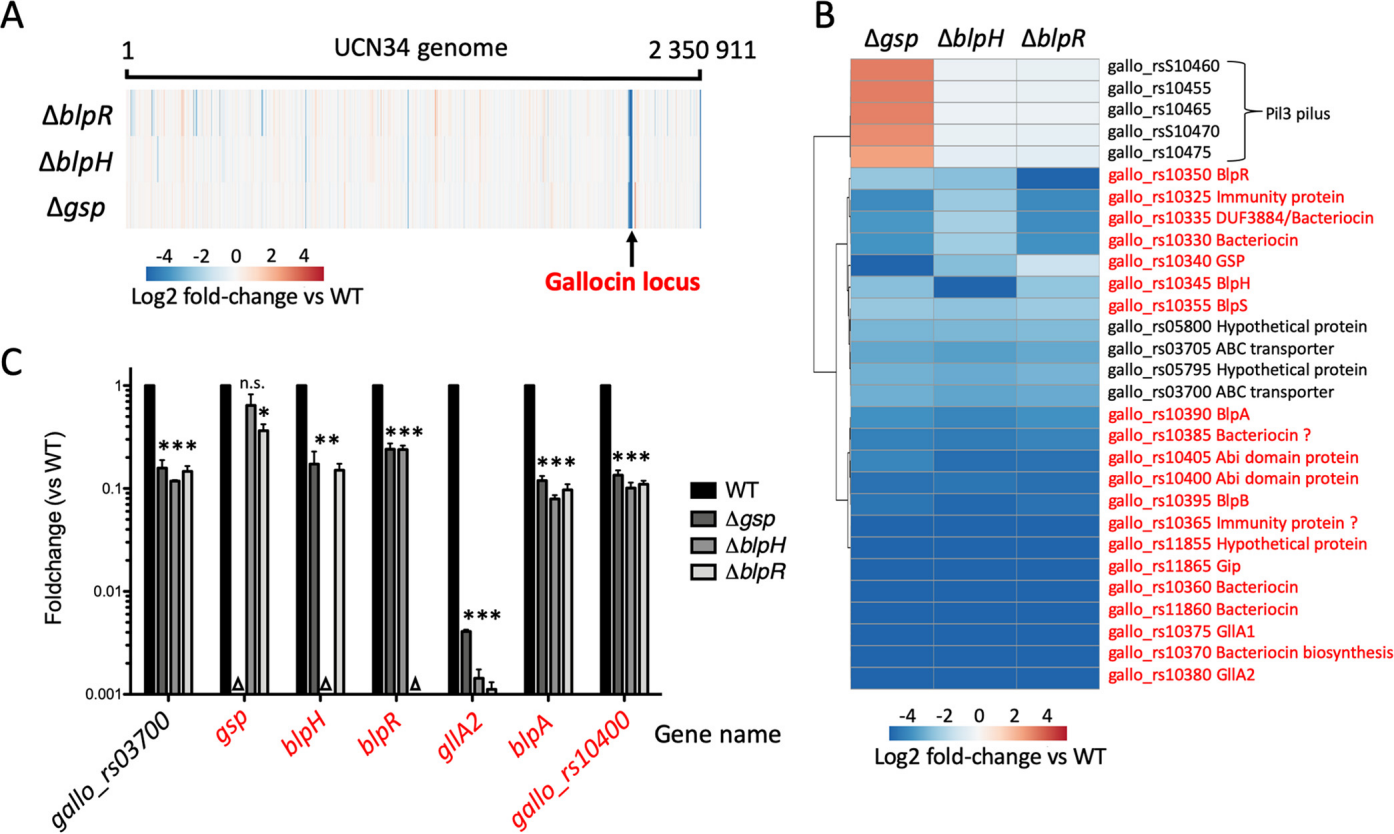
The whole regulon controlled by GSP/BlpHR. (A) Heat map representing the log_2_ fold change in mRNA abundance (determined by whole-transcriptome analysis) of all the genes along the UCN34 genome in Δ*gsp*, Δ*blpH*, and Δ*blpR* mutants compared to the parental S. gallolyticus subsp. *gallolyticus* UCN34 WT. (B) Analysis similar to that in panel A for selected genes whose log_2_ fold change in mRNA abundance was significantly greater than 2 or less than −2 in at least one mutant (see Materials and Methods). All the genes belonging to the gallocin locus are in red, along with the corresponding gene product. Gene product was determined either with genome annotation or by BLAST analysis. A question mark indicates that the result of the BLAST analysis was not found to be relevant. (C) qRT-PCR data showing the fold change in mRNA abundance in S. gallolyticus subsp. *gallolyticus* UCN34 Δ*gsp*, Δ*blpH*, and Δ*blpR* compared to the WT. The identity of each mutant was confirmed by the absence of transcript (triangles). Results are means and standard deviations (SD) from three independent cultures in triplicate. Asterisks represent statistical differences relative to WT strain UCN34. *, *P* < 0.05; **, *P* < 0.01; ***, *P* < 0.001; n.s., no significant difference as assessed by using ANOVA in R package version 1.4.2.

In order to identify genes potentially involved in the production, maturation, and secretion of gallocin and to uncover new genes potentially coregulated with gallocin genes, we performed a whole-transcriptome analysis of the S. gallolyticus subsp. *gallolyticus* UCN34 WT, Δ*blpR*, Δ*blpH*, and Δ*gsp* strains using total RNAs extracted from exponentially growing cultures. The transcriptional profiles of the three mutants were very similar, and their comparison with that of parental UCN34 WT shows that the main targets of the BlpRH regulatory system are the genes present in the gallocin locus whose expression is strongly lowered in the three mutants (Fig. 2A).

Selecting genes whose transcription is significantly different (log_2_ fold change, <−2 or >2; *P* value < 0.01) in at least one mutant compared to S. gallolyticus subsp. *gallolyticus* WT UCN34 showed that 24 genes were downregulated in the three mutants (Fig. 2B). Twenty of these 24 genes belong to the gallocin locus displayed in Fig. 1A (in red in Fig. 2B). These were (i) the regulatory module including *gsp*, *blpH*, and *blpR*, plus the upstream adjacent *blpS* gene, encoding a putative DNA binding protein; (ii) the two genes encoding the gallocin peptides (*gllA1* and *gllA2* [*gallo_rs10375* and *gallo_rs10380*]) and the putative immunity peptide (*gip* [*gallo_rs11865*]); (iii) the two genes (*blpAB*) encoding the ABC transporter whose role in the secretion of gallocin and GSP peptides is demonstrated in the accompanying paper (13); (iv) *gallo_rs10370*, which encodes a conserved protein with an undefined role in bacteriocin biosynthesis; (v) *gallo_rs10400* and *gallo_rs10405*, encoding Abi domain proteins; and (vi) several hypothetical genes (indicated by black arrows in Fig. 1A) that may encode putative bacteriocins and immunity peptides (*gallo_rs10325*/*10335*, *gallo_rs10360*/*10365*, *gallo_rs11860*, and *gallo_rs10385*). Beside the gallocin locus, only four genes clustered in two different loci were found to be downregulated in the three regulatory mutants. These were the adjacent genes *gallo_rs03700*/*gallo_rs03705*, encoding a putative ABC transporter, and *gallo_rs05795*/*gallo_rs05800*, encoding hypothetical proteins of unknown function. Of note, the strong upregulation of the Pil3 pilus operon in the *Δgsp* mutant (Fig. 2B) was caused by a phase variation event as described previously (17) (data not shown).

To validate the transcriptome analysis, qRT-PCR was performed on 7 representative genes (Fig. 2C). qRT-PCR results confirmed the downregulation of these genes in the absence of either GSP or BlpRH TCS. Transcription of the core gallocin *gllA* operon was more strongly reduced (>20-fold) than that of the other genes of the locus, such as those encoding the regulatory system (5-fold) and the ABC transporter (8-fold). It is worth noting that transcription of the *gsp* gene was only moderately altered in the Δ*blpH* and Δ*blpR* mutants compared to the UCN34 WT (fold change in Δ*blpH*, 0.159 by transcriptome analysis and 0.64 by qRT-PCR; fold change in Δ*blpR*, 0.4 by transcriptome analysis and 0.37 by qRT-PCR). Together, these results show that this regulatory system strongly activates the transcription of several genes involved in bacteriocin biosynthesis and also induces its own transcription, albeit at a lower level.

### Identification of a second regulator, BlpS, preventing transcriptional activation by BlpR.

**FIG 3:**
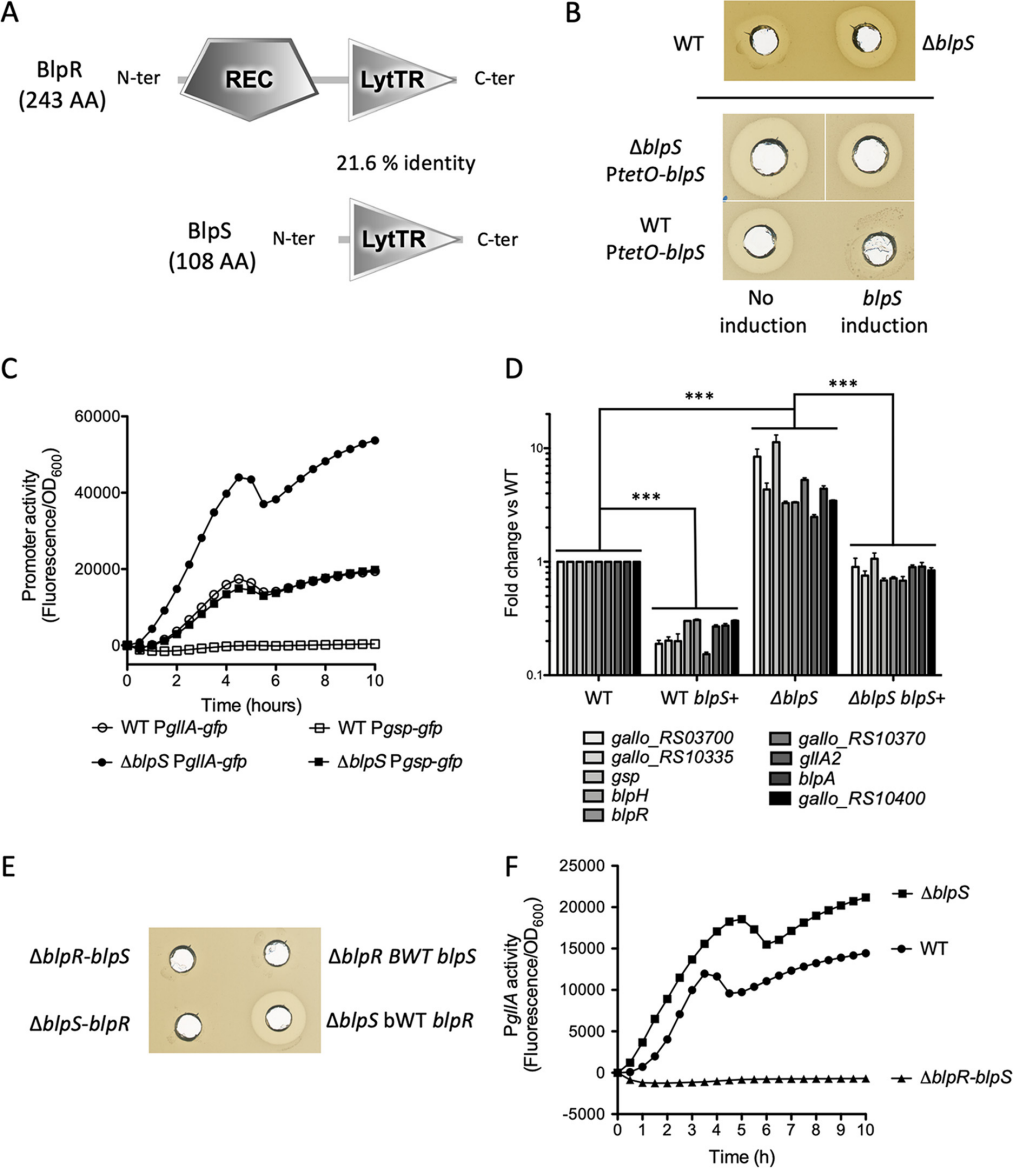
BlpS inhibits gallocin gene transcription. (A) SMART domains identified in BlpR and BlpS proteins. REC, *cheY*-homologous receiver domain; LytTR, LytTR DNA-binding domain. The percent identity between the two LytTR domains was determined using the Geneious alignment tool. (B) Agar diffusion assay showing gallocin activity in the culture supernatant against S. gallolyticus subsp. *macedonicus*. The strains tested were S. gallolyticus subsp. *gallolyticus* UCN34 WT and Δ*blpS* (top) and the same strains containing pTCVΩP*tetO*-*blpS* with or without induction of *blpS* expression with 200 ng/ml anhydrotetracycline (bottom). (C) Promoter activity of P*gllA* (circles) and P*gsp* (squares) during growth in S. gallolyticus subsp. *gallolyticus* WT and the Δ*blpS* mutant. One representative curve of three independent experiments is shown here for each condition. (D) qRT-PCR data showing the fold change in mRNA abundance between S. gallolyticus subsp. *gallolyticus* UCN34 pTCVΩP*tetO*-*blpS* (WT) and Δ*blpS* pTCVΩP*tetO*-*blpS* (Δ*blpS*). *blpS*+ indicates the induction of *blpS* transcription with 200 ng/ml anhydrotetracycline. Results are means and SD from three independent cultures carried out in triplicate. Statistical differences for each gene in the various groups were assessed using ANOVA in R package version 1.4.2. ***, *P* < 0.001. (E) Agar diffusion assay to test gallocin activity in the culture supernatant of S. gallolyticus subsp. *gallolyticus* UCN34 Δ*blpS* Δ*blpR* (deletion of *blpS* in Δ*blpR*), UCN34 Δ*blpR* Δ*blpS* (deletion of *blpR* in Δ*blpS*), and the respective bWT strains against S. gallolyticus subsp. *macedonicus*. (F) P*gllA* activity during growth in S. gallolyticus subsp. *gallolyticus* UCN34 WT, Δ*blpS* and Δ*blpR* Δ*blpS*. One representative curve of three independent experiments is shown for each condition.

The *blpRH* genes encode a typical TCS composed of a response regulator, BlpR, which contains a CheY-homologous phospho-receiver domain and a LytTR DNA binding domain (Fig. 3A), and a sensor histidine kinase, BlpH, with 5 transmembrane regions. A second regulatory gene encoding a putative DNA-binding protein consisting entirely of a LytTR DNA-binding domain was found immediately upstream of *blpRH* (Fig. 3A). We thus decided to test the role in gallocin production of this additional gene, designated *blpS*, which is likely cotranscribed with *blpRH*. A clean in-frame deletion of this gene was performed in S. gallolyticus subsp. *gallolyticus* UCN34 to avoid polar effects on the downstream *blpRH* genes. Interestingly, the Δ*blpS* strain produced about 4-fold more gallocin than the WT, as determined by serial dilution of the supernatant necessary to kill S. gallolyticus subsp. *macedonicus* prey strain, suggesting that BlpS represses gallocin gene expression. We then overexpressed *blpS* in the WT and Δ*blpS* mutant strains using an inducible expression vector (pTCVΩP*tetO*-*blpS*). As a prerequisite, we first demonstrated that the inducible promoter P*tetO* is functional in S. gallolyticus subsp. *gallolyticus* using *gfp* as a reporter gene (pTCVΩP*tetO*-*gfp*) (Fig. S2). We then showed that induction of *blpS* transcription leads to a decrease in gallocin production in both the Δ*blpS* and WT strains (Fig. 3B).

We next tested the effect of *blpS* deletion on the *gllA* and *gsp* promoters. Reporter plasmids in which *gfp* expression was placed under the control of the P*gllA* or P*gsp* promoters were introduced in UCN34 WT and Δ*blpS* strains. As shown in Fig. 3C, expression from the *gllA* and *gsp* promoters is strongly increased in the Δ*blpS* mutant compared to the WT.

To determine the impact of BlpS on the whole regulon controlled by the GSP/BlpHR module, we quantified by qRT-PCR the transcription levels of 9 different genes of this regulon, one located outside (*gallo_rs03700*) and eight within the gallocin genomic locus (*gallo_rs10335* [encoding a putative bacteriocin], *gsp*, *blpH*, *blpR*, *gallo_rs10370*, *gllA2*, *blpA*, and *gallo_rs10400*) in the WT and Δ*blpS* strains expressing *blpS* under the control of the inducible promoter P*tetO*. Expression of the 9 tested genes was increased in the Δ*blpS* mutant compared to the WT strain in the absence of inducer (Fig. 3D). Induction of *blpS* expression reduced the transcription levels of the 9 tested genes in both the WT and Δ*blpS* strains (Fig. 3D). Expression of *gsp* displayed the highest fold change between the WT and Δ*blpS* strains, with more than a 10-fold increase in the Δ*blpS* mutant. These results indicate that BlpS provides a negative feedback loop to control gallocin gene expression (Fig. 3D).

To establish the epistatic relationship of BlpR and BlpS on gallocin production, both genes were deleted, either by deleting the *blpS* gene in the Δ*blpR* mutant or the reverse. As shown in Fig. 3E and F, Δ*blpS* Δ*blpR* mutants were unable to produce gallocin, and consistently, the gallocin promoter P*gllA* was totally inactive in these mutants. Together, these results demonstrate that BlpR is epistatic over BlpS.

### Importance of gallocin expression for the killing of sensitive Enterococcus faecalis in gut-like conditions.

To assess experimentally the impact of gallocin production in relevant *in vivo* conditions, a bacterial competition assay was developed for the different S. gallolyticus subsp. *gallolyticus* mutants used in this study and two other gut bacteria: Enterococcus faecalis, which is sensitive to gallocin, and Escherichia coli *pks*^+^, which is resistant to gallocin. These competitions were performed in the gut microbiota medium at 37°C in a 5% CO_2_ incubator in order to mimic the conditions that the bacteria encounter in the host intestinal tract.

**FIG 4:**
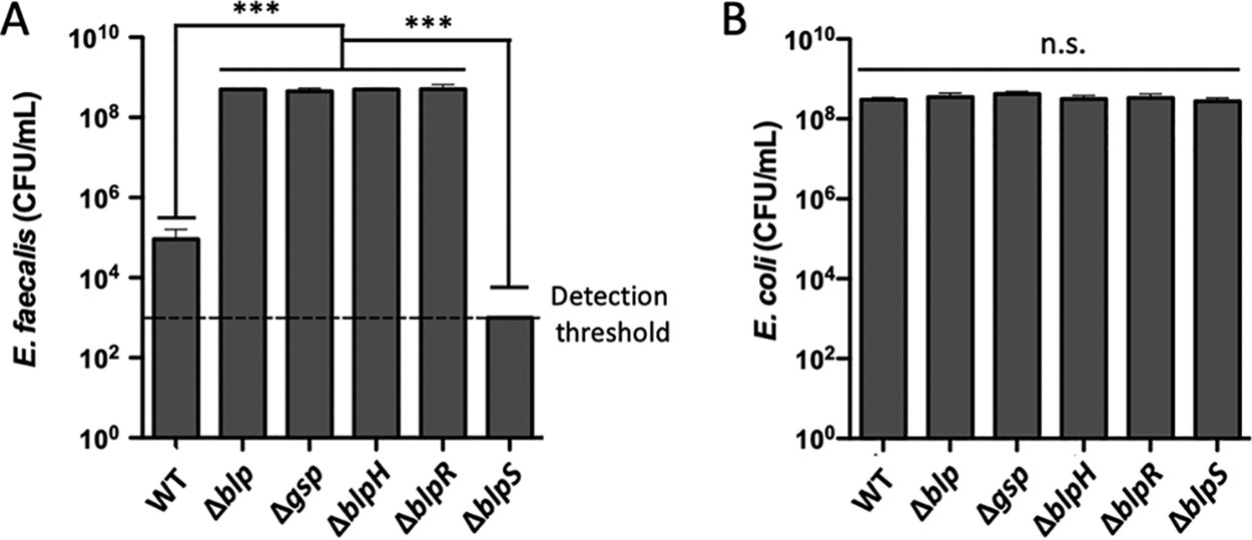
Overexpression of gallocin in *blpS* mutant allows better killing of E. faecalis in a competition experiment under gut-like conditions. (A) E. faecalis V583 counts after a 5-h competition against different strains of S. gallolyticus subsp. *gallolyticus*. (B) E. coli *pks*^+^, a strain resistant to gallocin used as a control. S. gallolyticus subsp. *gallolyticus* Δ*blp* is the mutant initially constructed (4), which has the three genes of the gallocin-encoding core operon deleted (Δ*gllA1* Δ*gllA2* Δ*gip*). Statistical differences for each strain were assessed using ANOVA in R package version 1.4.2. ***, *P* < 0.001; n.s., no statistically significant difference found.

After 5 h of competition, the number of E. faecalis CFU was about 5,000 times lower with S. gallolyticus subsp. *gallolyticus* WT than with the gallocin-defective mutants, confirming the inhibitory activity of gallocin under these conditions (Fig. 4A). This inhibitory effect on E. faecalis growth was even stronger with the *blpS* mutant, as we did not observe any E. faecalis CFU on the selective Entero agar plates after competition (the lower detection threshold in these experiments was considered to be 1,000 CFU/ml). Control competition experiments with gallocin-resistant gut E. coli *pks*^+^ did not show any variations in E. coli CFU with the various S. gallolyticus subsp. *gallolyticus* mutants (Fig. 4B). In some of these experiments, growth defects were observed when the number of S. gallolyticus subsp. *gallolyticus* WT or Δ*blpS* CFU per milliliter at the end of the competition experiment was compared to that of the gallocin-defective mutants (Δ*gsp*, Δ*blpH*, Δ*blpR*, and Δ*blp*). It suggests that gallocin production may have a biological cost under certain conditions, even if the results were not statistically significant.

### Identification of a consensus DNA motif upstream from genes controlled by the BlpRH TCS.

**FIG 5:**
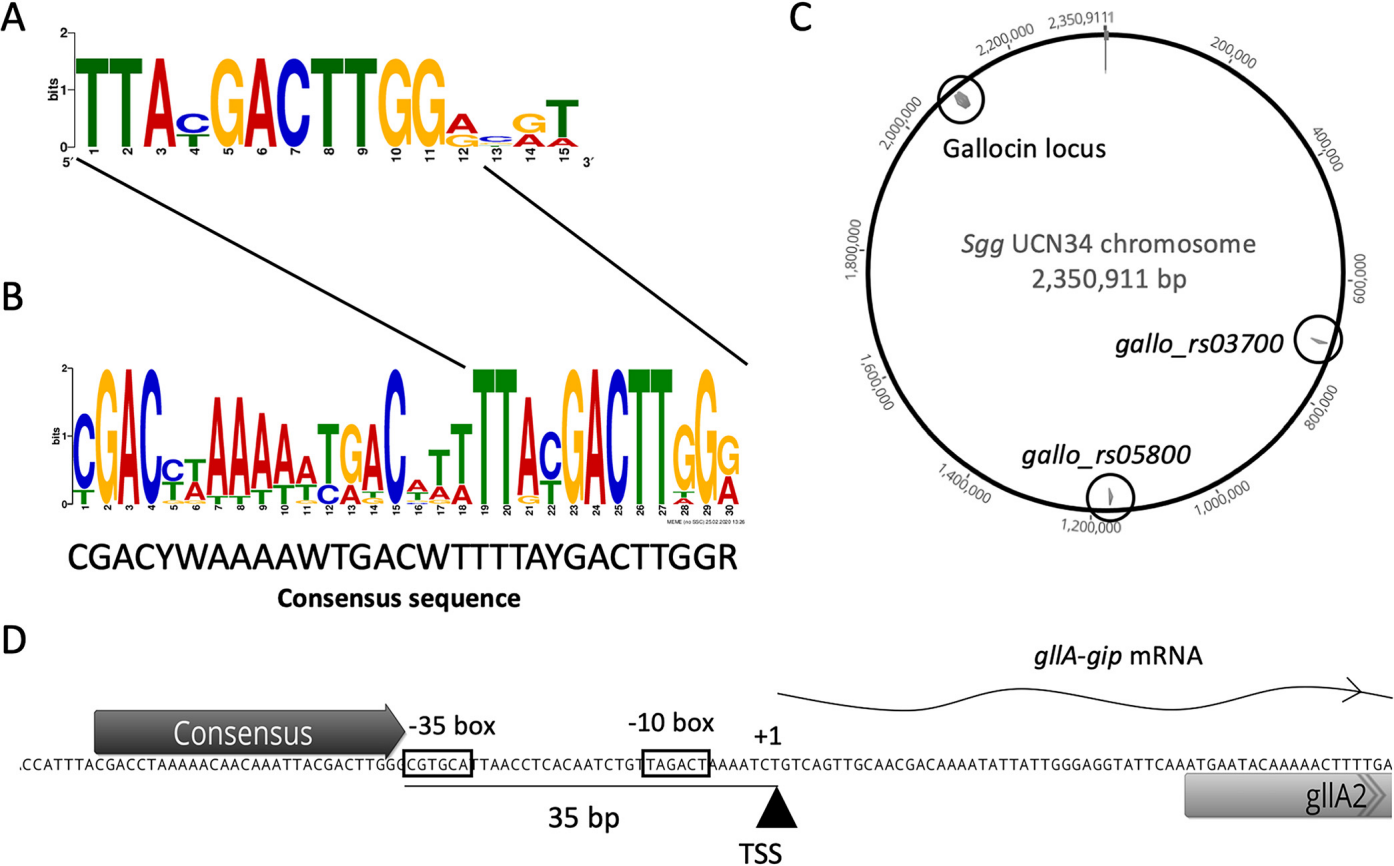
A conserved DNA motif is present upstream of all the genes regulated by GSP-BlpHR. (A) The 15-bp DNA motif obtained by alignment of the promoters of the regulatory system, the bacteriocin accessory protein gene, gallocin genes, the ABC transporter gene, and the Abi domain protein gene on https://weblogo.berkeley.edu/logo.cgi. (B) A 30-bp consensus sequence identified by MEME in the 12 putative promoters regulated by BlpHR. The initial 15-bp motif is located at the 3′ end of the larger consensus motif. (C) Mapping of the 30-bp consensus sequence on the S. gallolyticus subsp. *gallolyticus* chromosome (with a maximum of 6 mismatches). The consensus sequences are represented by arrowheads, and the name of the gene downstream of the consensus is given. (D) Determination of the transcription start site of *gllA* mRNA and localization of the conserved motif. Putative −10 (TAGACT) and −35 (CGTGCA) promoter boxes were assigned based on the location of the (+1) transcription start site according to the canonical procaryotic promoter sequence (TTGACA-X17-TATAAT).

We next searched for a conserved DNA motif acting as a putative binding site(s) in the promoter regions of the genes regulated by BlpR and BlpS. Our initial promoter sequence alignment of the gallocin locus genes (i.e., 250 bp upstream from the initiation codons) using Geneious software identified a conserved 15-bp motif (Fig. 5A). To improve the robustness of the 15-bp consensus sequence, promoters of the 12 putative operons regulated by BlpHR were analyzed with MEME software (http://meme-suite.org/tools/meme). A larger consensus sequence of 30 bp, including most nucleotides of the previously identified 15-bp motif (12 bp of 15), was identified in all regulated promoters (Fig. 5B). Mapping of this motif on the whole S. gallolyticus subsp. *gallolyticus* UCN34 chromosome showed that it is highly specific, as it is present only upstream from the operons in the gallocin locus, as well as the two other bicistronic loci, *gallo_rs03700* and *gallo_rs05800*, identified by the transcriptome analysis (Fig. 5C and 1A). This 30-bp consensus motif, which is a likely the binding site for BlpR and/or BlpS, contains 3 short repeats of 4 bp (C/TGAC). To properly map this motif in the P*gllA* promoter, we determined the transcription start sites (TSS) of the gallocin *gllA*-*gip* operon by RACE (rapid amplification of cDNA ends)-PCR. The 30-bp consensus motif lies just upstream of the −35 region of the operon promoter (Fig. 5D).

### Direct binding of BlpR and BlpS to various regulated promoters.

**FIG 6:**
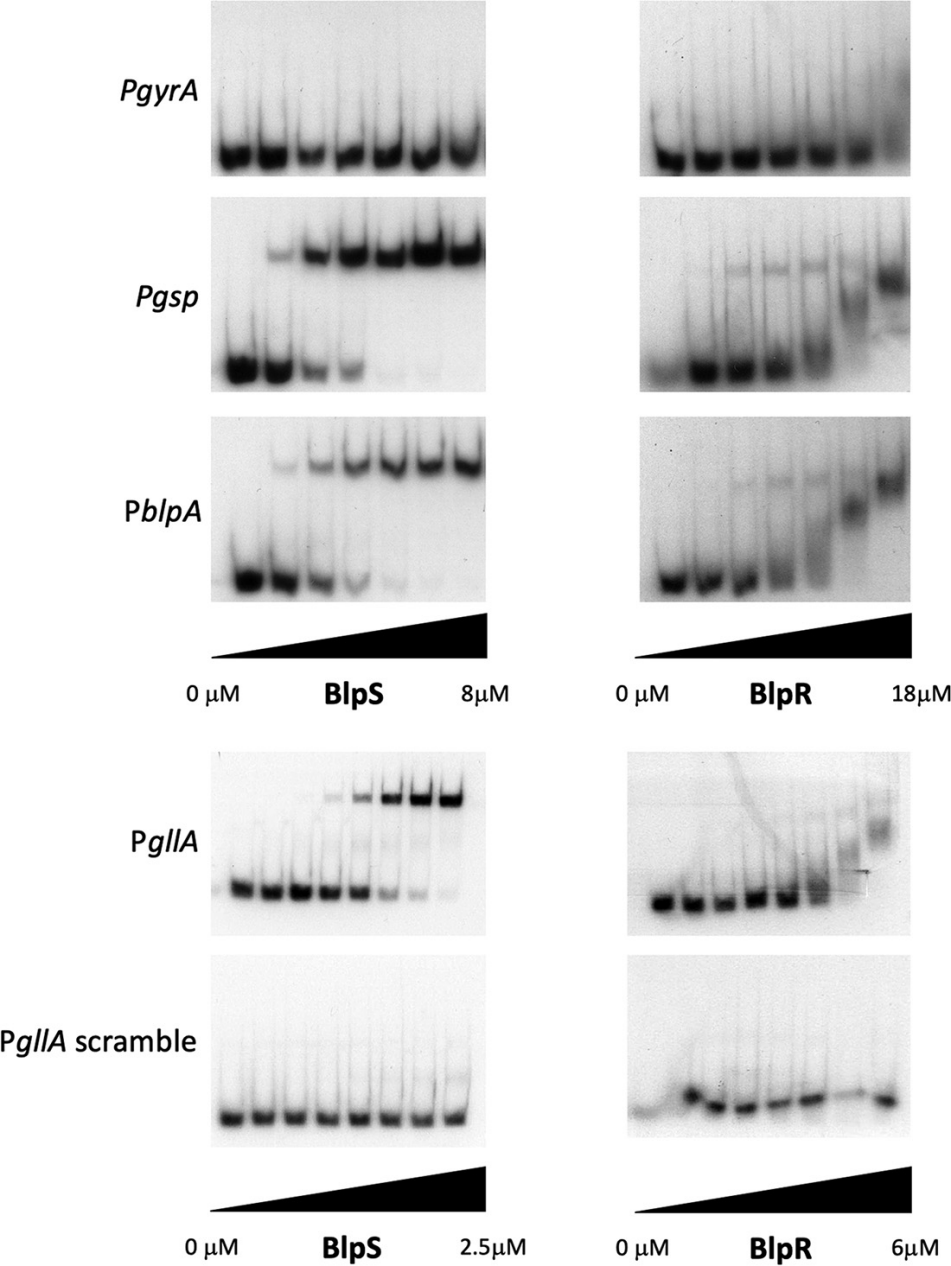
Binding of BlpR and BlpS to three regulated promoters. EMSA demonstrating the binding of BlpS and BlpR to the promoter regions of *gsp* (P*gsp*), *blpA* (P*blpA*), *gllA* (P*gllA*), and P*gllA* where the consensus sequence was randomly scrambled (P*gllA* scramble). P*gyrA* was used as a negative control. The full sequences of the various promoters are presented in Table 3. Serial 2-fold dilutions of the recombinant protein (from right to left) were incubated with purified radiolabeled promoters before migration. The leftmost band corresponds to migration of the promoter alone. All these experiments were carried out in the presence of 0.1 μg/μl poly(dI-dC) to prevent aspecific binding of proteins to DNA. Results of one representative EMSA of three independent experiments are shown for each condition.

To test the binding of BlpR and BlpS to the promoter regions that they control, electrophoretic mobility shift assays (EMSAs) were conducted on the regulated promoters P*gllA*, P*gsp*, and P*blpA* and on the P*gyrA* promoter as a negative control. BlpR and BlpS were produced as recombinant N-terminally histidine-tagged proteins in Escherichia coli BL21(DE3) and purified by immobilized metal ion affinity chromatography. The purified proteins migrated around their expected molecular masses (14.1 kDa and 29.3 kDa for 6×His-BlpS and 6×His-BlpR, respectively) and were detected by Western blotting using a His-tagged monoclonal antibody (Fig. S3A and B). Direct binding of recombinant BlpR and BlpS to the three regulated promoters, i.e., P*gllA*, P*blpA*, and P*gsp*, was observed by EMSA in a dose-dependent manner, while no binding to the control promoter P*gyrA* was detected (Fig. 6).

10.1128/mBio.03187-20.3 FIG S3 SDS-PAGE and Western blot analysis of purified histidine-tagged recombinant BlpR and BlpS proteins (A) Visualization of purified recombinant BlpR and BlpS, concentrated 10-fold, on Vivaspin columns (cutoff, 5,000 Da) by Coomassie staining and (B) Western blotting using primary anti-His-tag monoclonal antibody followed by a secondary fluorescent conjugated goat anti-mouse immunoglobulin antibody. The positions of BlpR and BlpS monomers are indicated with dark arrowheads. Download FIG S3, TIF file, 0.2 MB. Copyright © 2021 Proutière et al. 2021 Proutière et al. This content is distributed under the terms of the Creative Commons Attribution 4.0 International license.

**FIG 7:**
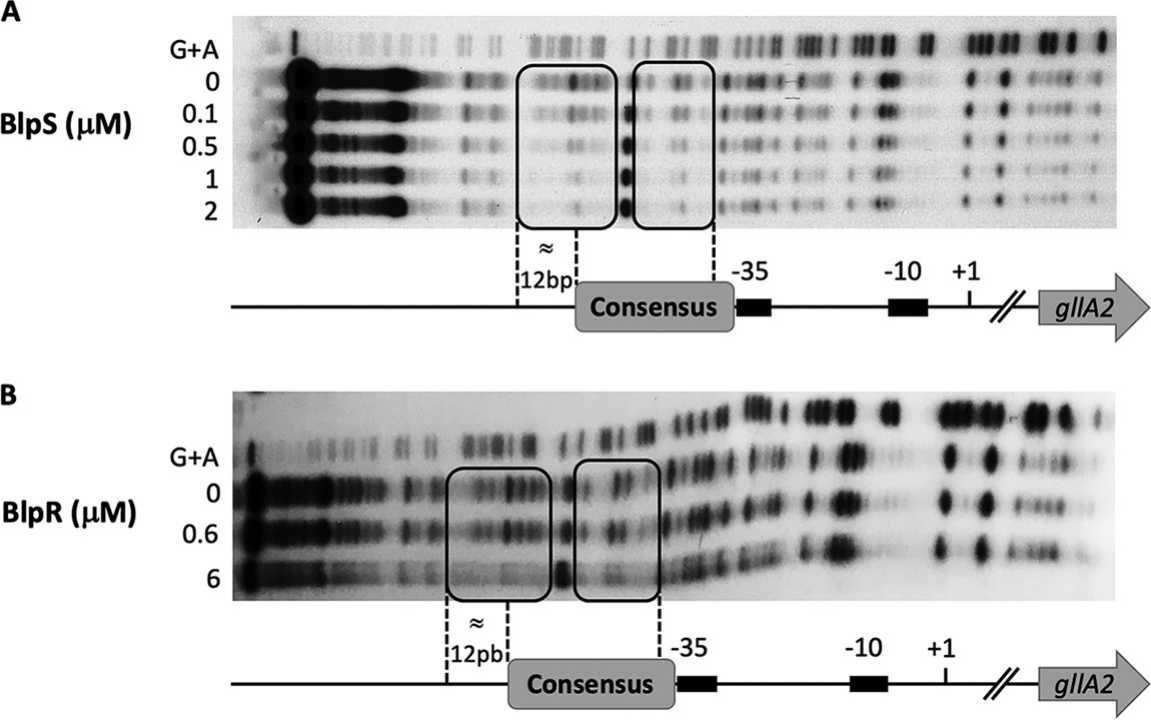
The binding site of BlpR and BlpS, mapped precisely by DNase I footprint experiment. Analysis of BlpR and BlpS footprint on the *gllA* promoter. The *gllA* promoter was incubated with increasing concentrations of BlpR or BlpS (indicated on the left) and digested with DNase. The sites protected from DNase by BlpR or BlpS binding are indicated by black squares. The sequence was determined by G+A sequencing and mapped on the *gllA* promoter. (A) Footprint with BlpS protein. (B) Footprint with BlpR protein.

To demonstrate that the identified 30-bp consensus sequence is the binding site of BlpR and BlpS, EMSAs were repeated on a P*gllA* promoter in which the 30-bp motif was scrambled (Fig. S4). As shown in Fig. 6, binding of both BlpR and BlpS was completely abolished. To define precisely the binding site of these two regulators, footprint assays were carried out with purified BlpR and BlpS on *gllA* and *blpA* promoters. As expected from the EMSAs, BlpS clearly binds and protects DNA on the identified consensus sequence in *gllA* and *blpA* promoters (Fig. 7A; Fig. S5). The footprint was even larger than the consensus sequence and includes about 12 bp upstream of the consensus. Interestingly, the putative −35 box of *gllA* appears free in the presence of BlpS, suggesting that BlpS-mediated inhibition proceeds through competition with BlpR rather than inhibition of RNA polymerase binding by sequestration of the −35 motif. Consistent with this hypothesis, the BlpR binding site is apparently very similar to that of BlpS (Fig. 7B). However, footprint experiments with BlpR were more difficult to carry out, probably because BlpR appeared very unstable.

10.1128/mBio.03187-20.4 FIG S4 Alignment between P*gllA* and P*gllA*-scr. Alignment of the P*gllA*-scrambled promoter on the WT promoter region around the consensus 30-bp motif. Download FIG S4, TIF file, 0.2 MB. Copyright © 2021 Proutière et al. 2021 Proutière et al. This content is distributed under the terms of the Creative Commons Attribution 4.0 International license.

10.1128/mBio.03187-20.5 FIG S5 BlpS binding on *blpA* promoter. Analysis of BlpS footprint on *blpA* promoter. The *blpA* promoter was incubated with increasing concentration of BlpS (indicated on the left) and digested by DNase. The sites protected from DNase by BlpS binding are indicated by black squares. The sequence was determined by G+A sequencing and mapped on the *blpA* promoter. Download FIG S5, TIF file, 0.3 MB. Copyright © 2021 Proutière et al. 2021 Proutière et al. This content is distributed under the terms of the Creative Commons Attribution 4.0 International license.

### Phosphorylated BlpR binds to *gll*A promoter with a higher affinity than non-phosphorylated BlpR.

**FIG 8:**
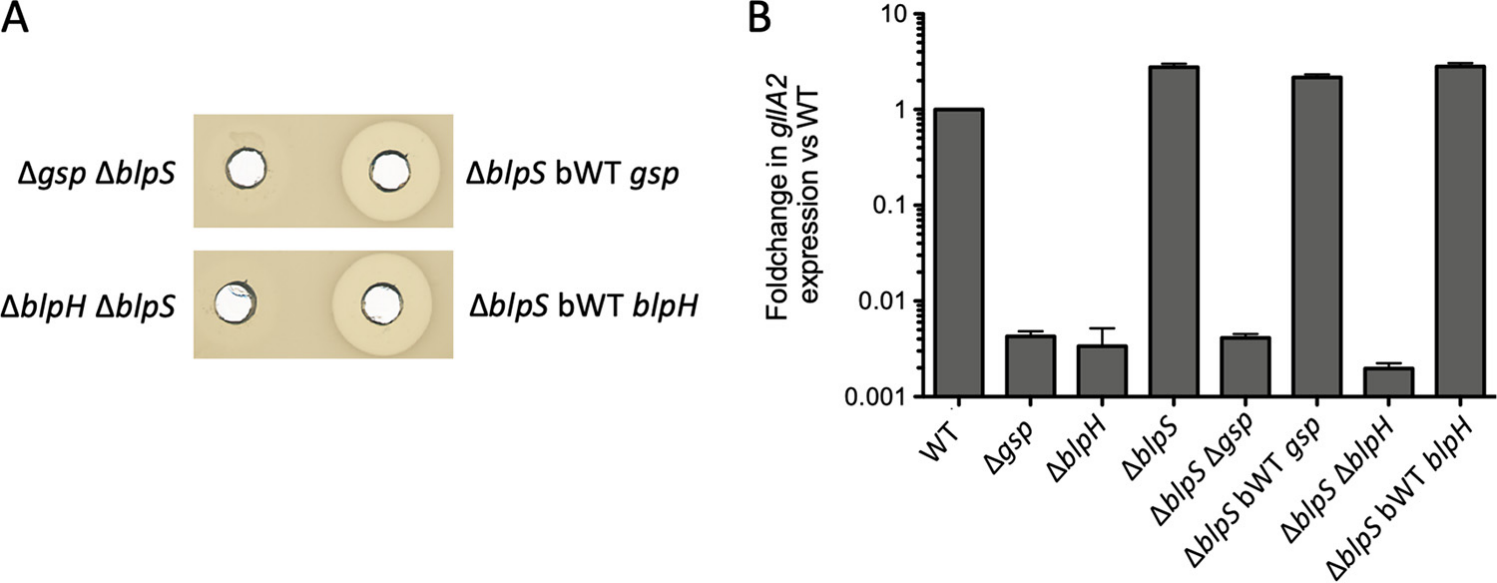
Genetic evidence demonstrating that non-phosphorylated BlpR cannot activate gallocin (*gllA2*) gene transcription. (A) Agar diffusion assay to assess gallocin activity in the culture supernatant against S. gallolyticus subsp. *macedonicus*. S. gallolyticus subsp. *gallolyticus* UCN34 Δ*blpS* Δ*gsp*, Δ*blpS* bWT *gsp*, Δ*blpS* Δ*blpH*, and Δ*blpS* bWT *blpH* strains were tested. (B) qRT-PCR data showing the fold change in mRNA abundance of the *gllA2* gene between S. gallolyticus subsp. *gallolyticus* UCN34 WT and S. gallolyticus subsp. *gallolyticus* mutants.

*In vitro* phosphorylation of BlpR using acetyl phosphate as a nonspecific phosphate donor increased its DNA binding affinity (Fig. S6). Since the putatively unphosphorylated form of BlpR was still able to bind *gllA* promoter (Fig. 6), we wondered if the role of BlpS could be to prevent the binding of unphosphorylated BlpR, while the phosphorylated form of BlpR could outcompete BlpS. To test this hypothesis, we constructed Δ*blpS* Δ*gsp* and Δ*blpS* Δ*blpH* mutants in S. gallolyticus subsp. *gallolyticus* UCN34 by deleting the *gsp* and *blpH* genes, respectively, in UCN34 Δ*blpS*. In these double mutants, BlpS is absent and BlpR should be unphosphorylated because either GSP or its associated histidine kinase BlpH is absent. The supernatants of these two mutants were not active against S. gallolyticus subsp. *macedonicus*, suggesting that unphosphorylated BlpR cannot activate gallocin expression even in the absence of the repressor BlpS (Fig. 8A). To validate this result at the transcriptional level, qRT-PCR experiments were carried out, and they showed that *gllA2* transcription is reduced at the same level in these double mutants as it is in in Δ*gsp* and Δ*blpH* strains (Fig. 8B). Taken together, these results demonstrate that only the phosphorylated form of BlpR can activate gallocin transcription and that BlpS competes with phosphorylated BlpR to reduce gallocin expression.

10.1128/mBio.03187-20.6 FIG S6 BlpR phosphorylation increases its DNA binding affinity. EMSA demonstrating the binding of BlpR, phosphorylated by acetyl phosphate (BlpR-P) or not (BlpR), to the promoter region of *gllA*. Download FIG S6, TIF file, 0.2 MB. Copyright © 2021 Proutière et al. 2021 Proutière et al. This content is distributed under the terms of the Creative Commons Attribution 4.0 International license.

## DISCUSSION

S. gallolyticus subsp. *gallolyticus* belongs to group D streptococci, a large group of phenotypically diverse bacteria known as the Streptococcus bovis/Streptococcus equinus complex (SBSEC), which consist of safe-graded bacteria used in food fermentation, commensal bacteria of the gut, and opportunistic pathogens in both humans and animals (18). S. gallolyticus subsp. *gallolyticus* is a commensal inhabitant of the rumens of herbivores, a complex ecological habitat harboring several thousand bacterial species. In humans, it is an opportunistic pathogen causing septicemia and endocarditis in elderly persons. Association between S. gallolyticus subsp. *gallolyticus* infections and underlying colon neoplasia has been reported by clinicians since the 1950s (2). Recently, we showed that S. gallolyticus subsp. *gallolyticus* strain UCN34 takes advantage of tumoral conditions to colonize the mouse colon (4). S. gallolyticus subsp. *gallolyticus* produces and secretes a specific bacteriocin, named gallocin, whose antimicrobial activity is potentiated by increased levels of secondary bile salts found in colonic neoplasia to inhibit the growth of closely related enterococcus commensals, thus creating a colonization niche for S. gallolyticus subsp. *gallolyticus* under tumoral conditions (4).

Gallocin is encoded by two genes, recently renamed *gllA1* and *gllA2*, which are absent from the most closely related bacteria belonging to the SBSEC, including S. gallolyticus subsp. *macedonicus*. Another gallocin variant was recently reported in an S. gallolyticus subsp. *gallolyticus* milk isolate and named gallocin D (15). Gallocin is a class II bacteriocin, and members of this family are widespread among lactic acid bacteria, including streptococci. These molecules are usually directed against closely related bacteria competing within the same environment. The genetic locus encoding gallocin in S. gallolyticus subsp. *gallolyticus* UCN34 is complex and shares similarities with other prototypical class II bacteriocin loci with genes encoding a putative immunity peptide, a dedicated ABC transporter, several other putative bacteriocins, and a regulatory system (7, 19).

In this work, we demonstrated that gallocin production in S. gallolyticus subsp. *gallolyticus* is induced by a secreted peptide named GSP (for “gallocin-stimulating peptide”) through the activation of a dedicated TCS composed of BlpH, a putative membrane histidine kinase, and BlpR, a putative cytoplasmic response regulator. Using a GFP-based reporter plasmid to monitor gallocin promoter (P*gllA*) activity, we showed that synthetic GSP activates gallocin promoter in a dose-dependent manner. GSP was shown to be secreted through the gallocin ABC transporter (designated BlpAB). A structure-function analysis of the GSP peptide demonstrated the importance of its C-terminal half (13). Since bacteriocin production has been linked to natural competence in various streptococci, including Streptococcus pneumoniae, S. mutans, and S. thermophilus, we looked at competence induction in S. gallolyticus subsp. *gallolyticus* UCN34 using a reporter plasmid in which the *comX* promoter was cloned upstream of the *gfp*. We showed that P*comX* is induced by XIP, the mature ComS peptide, in agreement with previous results (20). However, no P*comX* induction was observed using the GSP peptide (Fig. S7).

10.1128/mBio.03187-20.7 FIG S7 XIP but not GSP activates the *comX* promoter. Promoter activity of *comX* (P*comX*) in S. gallolyticus subsp. *gallolyticus* UCN34 (pTCVΩP*comX*-*gfp*) in the presence of XIP (1 mM) or GSP (20 nM) in M9Y medium (A) or in THY medium (B). Download FIG S7, TIF file, 0.5 MB. Copyright © 2021 Proutière et al. 2021 Proutière et al. This content is distributed under the terms of the Creative Commons Attribution 4.0 International license.

Our transcriptome sequencing (RNA-seq) data revealed that transcription of five other putative bacteriocin genes was coinduced with gallocin genes. Only one of these has a double glycine motif in the N terminus, similar to the gallocin peptides, while others have very different amino acid sequences (one being very rich in positively charged amino acids). Only two additional operons encoding an ABC transporter and hypothetical proteins, located elsewhere in UCN34 genome, were coinduced with the gallocin locus.

We also uncovered the role of a second regulatory protein named BlpS which represses all the genes activated by GSP/BlpRH. This small 108-amino-acid (aa) protein consists almost entirely of a LytTR DNA-binding domain. Most proteins containing a LytTR domain studied previously also contain an additional phospho-acceptor domain typical of TCS regulators (Interpro domain IPR007492) (21). Of note, two transcriptional regulators whose architecture is similar to that of BlpS were identified in S. mutans. However, these regulators are in operon with a transmembrane protein which inhibits their activity (22, 23). Thus, BlpS differs from these so-called LytTR regulatory systems (LRS) as it forms an operon with a classical TCS that it antagonizes. An *in silico* analysis revealed that 15,409 of the 80,096 LytTR-type regulators (Uniprot database) contained only this functional domain, and of these, 1,565 have a size similar to that of BlpS (between 100 and 120 amino acids). These proteins were found both in Gram-negative and Gram-positive bacteria. Among them, the homologous BlpS protein of the *blp* locus of Streptococcus pneumoniae was found. This *blp* locus, which encodes several bacteriocins named pneumocins, displays an organization highly similar to that of gallocin locus (24). We therefore speculate that *blpS* gene of S. pneumoniae potentially encodes a negative regulator of pneumocin production.

To define precisely the respective role of BlpR and BlpS in regulation, we constructed a Δ*blpR* Δ*blpS* mutant. This mutant did not produce gallocin, showing that BlpR is necessary for transcriptional activation of gallocin genes even in the absence of the repressor BlpS. Then, we showed by EMSA and by DNA footprinting that both BlpR and BlpS bind directly on the same consensus sequence that is present in all the promoter regions of the genes whose transcription is activated by BlpR (Fig. 6 and 7). Altogether, our results suggest that BlpS-mediated inhibition occurs through direct competition with BlpR at the same binding site.

**FIG 9:**
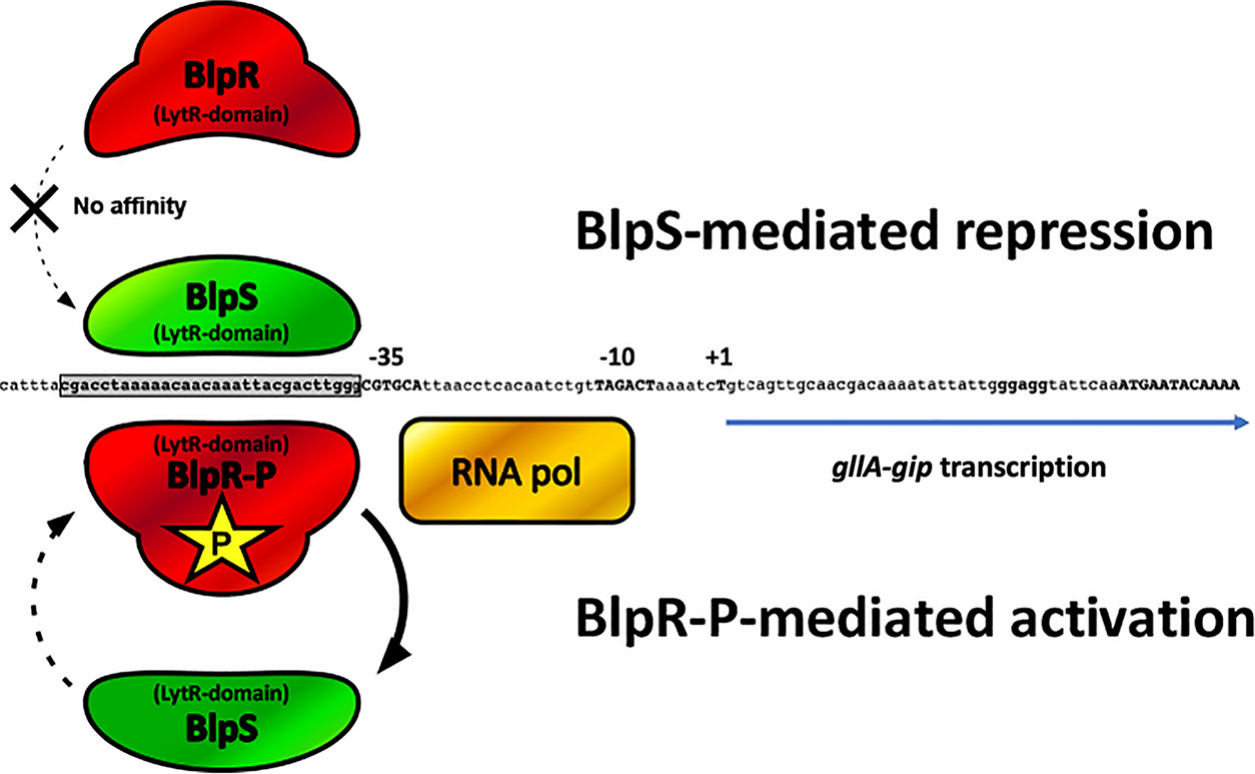
Hypothetical model of transcription regulation by BlpR and BlpS. At low cell density, i.e., in the absence of GSP or at low concentrations of GSP, BlpR is not phosphorylated by BlpH and hence has no affinity for the conserved binding motif located in the promoter of gallocin genes, which is occupied by the BlpS repressor. At high cell density, the GSP concentration is sufficient to induce BlpH-mediated phosphorylation of BlpR. Phosphorylated BlpR (BlpR-P) outcompetes BlpS, resulting in RNA polymerase recruitment and transcription of gallocin genes. The antagonistic effect of BlpS and BlpR-P controls the level of expression of the gallocin genes, which is shut down when GSP concentration decreases.

Although the recombinant BlpR purified from E. coli is presumably non-phosphorylated, it binds the tested promoters, albeit less efficiently than its phosphorylated form (Fig. S6). We thus hypothesized that the role of BlpS role was to prevent transcription activation by unphosphorylated BlpR. However, this possibility was ruled out in S. gallolyticus subsp. *gallolyticus* UCN34 through careful analysis of Δ*gsp* Δ*blpS* and Δ*blpH* Δ*blpS* double mutants, which were both unable to phosphorylate BlpR. In these mutants, no activation of *gllA2* transcription could be detected (Fig. 8). We thus propose the following working model to explain gallocin regulation through GSP-BlpRH-BlpS (Fig. 9). At low cell density, BlpR is unphosphorylated and cannot activate transcription, while BlpS binds to the promoters of the genes involved in gallocin production to block their transcription. At higher cell density, sufficient amounts of GSP are present to induce BlpH-mediated phosphorylation of BlpR, which, in turn, competes with BlpS to bind to the promoter region and trigger transcription of gallocin genes. The role of BlpS is likely to reduce the overactivation of the GSP-BlpRH system to prevent self-toxicity or reduce the metabolic costs associated with gallocin production and to rapidly shut down its synthesis when the concentration of inducer decreases.

In conclusion, we identified here an atypical four-component system involved in the regulation of bacteriocin production in S. gallolyticus subsp. *gallolyticus* UCN34, which could represent a new prototype of bacteriocin regulation. Bacteria have developed complex regulatory systems to control bacteriocin production in order to reduce its fitness cost. Indeed, we previously showed that the Δ*blp* mutant, which does not produce gallocin, colonizes better than its S. gallolyticus subsp. *gallolyticus* WT counterpart in the nontumoral murine intestinal tract. Although the Δ*blpS* mutant, which overproduced gallocin, did not exhibit a significant growth defect *in vitro*, it remains possible that the increased production of gallocin could have an impact on its fitness *in vivo*, where nutrients are limited.

Finally, since gallocin is particularly active under tumoral conditions, it will be important in future studies to see if some tumoral metabolites could induce gallocin transcription *in vivo*.

## MATERIALS AND METHODS

### Cultures, bacterial strains, plasmids, and oligonucleotides.

*Streptococcus* strains used in this study were grown at 37°C in Todd-Hewitt broth supplemented with yeast extract 0.5% (THY) in standing filled flasks. When appropriate, 10 μg/ml of erythromycin was added for plasmid maintenance.

Plasmid construction was performed by PCR amplification of the fragment to insert in the plasmid with Q5 High-Fidelity DNA polymerase (New England Biolabs), digestion with the appropriate FastDigest restriction enzyme (Thermo Fisher), ligation with T4 DNA ligase (New England Biolabs), and transformation in commercially available TOP10 competent E. coli (Thermo Fisher). E. coli transformants were cultured in Miller’s LB supplemented with 150 μg/ml erythromycin (for pG1-derived plasmids) or 50 μg/ml kanamycin (for pTCV-derived and pET28a plasmids). Verified plasmids were electroporated in S. agalactiae NEM316 and mobilized from NEM316 to S. gallolyticus subsp. *gallolyticus* UCN34 by conjugation as described previously (25). All the strains used and constructed in this study are listed in Table 1, and the primers are shown in Table 2.

**TABLE 1:** Strains used in this study

Strain	Description	Reference
NEM2431	Streptococcus gallolyticus subsp. *gallolyticus* UCN34	14
NEM2824	Enterococcus faecalis V583	31
	E. coli *pks*^+^	32
NEM4838	UCN34 Δ*blp*	4
NEM4522	Escherichia coli TOP10 pTCVΩP*gllA*-*gfp*	This study
NEM4850	UCN34 Δ*gsp*	This study
NEM4853	UCN34 Δ*gsp* pTCVΩP*gllA*-*gfp*	This study
NEM4858	UCN34 Δ*blpH*	This study
NEM4883	UCN34 Δ*blpH* pTCVΩP*gllA*-*gfp*	This study
NEM4855	UCN34 Δ*blpR*	This study
NEM4872	UCN34 Δ*blpR* pTCVΩP*gllA*-*gfp*	This study
NEM4851	UCN34 bWT *gsp*	This study
NEM4859	UCN34 bWT *blpH*	This study
NEM4856	UCN34 bWT *blpR*	This study
NEM5097	UCN34 Δ*blpS*	This study
NEM5098	UCN34 bWT *blpS*	This study
	UCN34 Δ*blpS* pTCVΩP*gllA*-*gfp*	This study
NEM5171	E. coli pTCVΩP*gsp*-*gfp*	This study
NEM5172	UCN34 pTCVΩP*gsp*-*gfp*	This study
NEM5218	UCN34 Δ*blpS* pTCVΩP*gsp*-*gfp*	This study
NEM5217	E. coli GM48 pTCVΩP*tetO*-*blpS*	This study
NEM5202	UCN34 pTCVΩP*tetO*-*blpS*	This study
NEM5204	UCN34 Δ*blpS* pTCVΩP*tetO*-*blpS*	This study
NEM5227	UCN34 Δ*blpR* Δ*blpS*	This study
NEM5228	UCN34 bWT *blpR* Δ*blpS*	This study
NEM5229	UCN34 Δ*blpS* Δ*blpR*	This study
NEM5230	UCN34 bWT*blpS* Δ*blpR*	This study
NEM5394	UCN34 Δ*blpS* Δ*gsp*	This study
NEM5397	UCN34 Δ*blpS* bWT *gsp*	This study
NEM5425	UCN34 Δ*blpS* Δ*blpH*	This study
NEM5427	UCN34 Δ*blpS* bWT *blpH*	This study
NEM5214	E. coli BL21(DE3) pet28aΩ*blpR*	This study
NEM5216	E. coli BL21(DE3) pet28aΩ*blpS*	This study
NEM5255	E. coli TOP10 pTCVlacΩP*gsp*	This study
NEM5258	E. coli TOP10 pTCVlacΩP*blpA*	This study
NEM5257	E. coli TOP10 pTCVlacΩP*gllA*	This study
NEM5456	E. coli TOP10 pTCVlacΩP*gllA*-scramble	This study
NEM5287	E. coli TOP10 pTCVlacΩP*gyrA*	This study
NEM4875	UCN34 pTCVΩP*comX*-*gfp*	This study

**TABLE 2:** Primers used in this study

Use and/or construct	Sequence (5′–3′)^*a*^
Mutant construction	
*gsp* deletion	TTCT**GAATTC**CCGTCGTAAATTCTAACT
	ATTTTTGGTTTAAGCGTGTTTTATAAACCTCCTTAG
	CTAAGGAGGTTTATAAAACACGCTTAAACCAAAAAT
	TTCTG**GGATCC**TTTATTCAGCATAGTCGC
*blpH* deletion	TTCT**GAATTC**GTTTTACTGATGCCACTG
	AGATGAGGTGAAAAGCTACTGAAATAGTCGAATGAT
	ATCATTCGACTATTTCAGTAGCTTTTCACCTCATCT
	TTCTG**GGATCC**CAAGGAATTGATGTCGCT
*blpR* deletion	TTCT**GAATTC**CTTCCATACCTGTTAGAA
	GATTTTTAGAGGAGATTTGGTGAAAAGCTAATGAGC
	GCTCATTAGCTTTTCACCAAATCTCCTCTAAAAATC
	TTCTG**GGATCC**ATCTTTTCTCTAATATGG
*blpS* deletion	TTCT**GAATTC**CAGCAGGAGAGGTTTCAA
	GGAGGGAATAATGATTTATGGACTGATTTTTAGAGGAG
	CTCCTCTAAAAATCAGTCCATAAATCATTATTCCCTCC
	TTCT**GGATCC**TTGGCGAAAAAATCCTCG
Promoters	
*gsp* promoter	TTCT**GAATTC**TTTTCAACTCATAACGAA
	TTCT**GGATCC**TTTTATAAACCTCCTTAG
*gllA* promoter	TTCT**GAATTC**GGTCCCAATCTCCCTT
	TTCT**GGATCC**TTGAATACCTCCCAAT
qPCR	
*rpoB*	CACCGTACACGTCGTAGC
	CCGTAAAGTTTGTAATCG
*gallo_rs03700*	CGAGGTATCCTTTTGTGT
	GGTATCACTCATAATTCC
*gallo_rs10335*	TTCCAACCTATACGCATG
	AGCTTGTTGAATGAAGGC
*gsp*	GATGACAGAAAAAATGTT
	GTGTTTAGTAGGCTTATG
*blpH*	ATGTTAGAGGAGCAAAGC
	ACTCTCTATAACCCATGG
*blpR*	AGAGGTGTTTAGTTCCGC
	CTACTAACGCTTGGTAGG
*blpS*	GGCTATTGACGATATCCT
	GTCGCTGCTCTCTATCCA
*gallo_10370*	CTGGCTCATCTGATGTGTC
	GTGCCTACAACTGAAACGA
*gllA2*	GAAGGTGGTTACAGCAAGACAG
	CTACACAAGTAGCCCCACCAC
*blpA*	CTCGCTGGCTCATTTGAG
	GCGGGAGTTTGCCTTCTT
*gallo_rs10400*	GGCGTTTTTGGTAGCATTA
	CAGCAGATAGTAAGCAATC
Overexpression	
*blpS*	TTCT**CTGCAG**CTCCTCTAAAAATCAGTC
	TTCT**GGATCC**GTTATTGGAGGGAATAATG
RACE-PCR	
*gllA*	TACACCCGCCAATAGCAG
	CCACCACTAATTGTTTGC
His-tagged BlpR and BlpS	
BlpR	TTCT**GGATCC**TCACCTCATCTCATTTAA
	TTCT**CATATG**ATGTTAGATATTTATGTA
BlpS	TTCT**GGATCC**TCAGTCATTTGAGATGAT
	TTCT**GCTAGC**ATGAAATATTTTAAATTTAC
EMSA promoters	
*gsp*	TTCT**GGATCC**TCATTTTTATAAACCTCC
	TTCT**GAATTC**GATGGCTTGGACTTTTTC
*blpA*	TTCT**GGATCC**CTTCTCATAACCTTTCCC
	TTCT**GAATTC**TTTGGAAGAATGGTAAAG
*gyrA*	TTCT**GGATCC**TAAGGAAAAACACTCCTT
	TTCT**GAATTC**TAAGTGAGATATGTCACG
*gllA*	TTCT**GGATCC**ATAATATTTTGTCGTTGC
	TTCT**GAATTC**ACGGTCAAAAAATCATGA
pTCV-lac primers	
VlacE	GAGTCAAAATAGATATGAACAAATG
VlacB	GCATTAGTGTATCAACAAGCTGGGG

a Restriction sites are in bold.

### Construction of markerless deletion mutants in S. gallolyticus subsp. *gallolyticus* UCN34.

In-frame deletion mutants were constructed as described previously (25). Briefly, the 5′ and 3′ regions flanking the region to delete were amplified and assembled by splicing by overlap extension PCR and cloned into the thermosensitive shuttle vector pG1. Once transformed in UCN34, the cells were cultured at 38°C with erythromycin to select for the chromosomal integration of the plasmid by homologous recombination. About 4 single crossover integrants were serially passaged at 30°C without antibiotic to facilitate the second event of homologous recombination and excision of the plasmid, resulting in either gene deletion or reversion to the WT (bWT). In-frame deletions were identified by PCR and confirmed by DNA sequencing of the chromosomal DNA flanking the deletion.

### Gallocin production assays.

Briefly, one colony of the indicator organism, S. gallolyticus subsp. *macedonicus*, was resuspended in 2 ml THY, grown to exponential phase, and poured onto a THY agar plate; the excess liquid was removed, and the plate was left to dry under a hood for about 20 min. Using sterile tips, 5-mm-diameter wells were dug into the agar. Each well was then filled with 80 μl of filtered supernatant from 5-h cultures (stationary phase) of S. gallolyticus subsp. *gallolyticus* WT or mutant strains and supplemented with Tween 20 (0.1% final concentration). Inhibition rings around the wells were observed the following morning after overnight incubation at 37°C.

### Monitoring promoter activity using a fluorescent reporter.

Promoter sequences of genes encoding gallocin (P*gllA*) or GSP (P*gsp*) were amplified with overhanging EcoRI and BamHI sites and cloned into the reporter pTCVΩ*gfp* vector upstream from the *gfp* gene to control its expression (pTCVΩP*gllA*-*gfp*; pTCVΩP*gsp*-*gfp*). Bacteria containing the plasmid were inoculated at an initial optical density at 600 nm (OD_600_) of 0.1 from fresh agar plates in 200 μl of medium in 96-well black plates. Due to the high autofluorescence of the THY medium, we switched to M9 medium supplemented with 0.5% yeast extract and 0.2% glucose (M9Y). If needed, synthetic GSP (from Genecust) was added to the medium at time zero. Promoter activity was then followed by continuous measurement of the growth and GFP fluorescence (one measurement every 30 min during 10 h) with the Synergy2 multidetection microplate reader (Biotek). Promoter activity was then estimated by dividing the fluorescence value by the OD_600_ value for each time point.

### Induction of the P*tetO* promoter in S. gallolyticus subsp. *gallolyticus*.

Using the reporter plasmid pTCVΩP*tetO*-*gfp*, we defined the minimal concentration of anhydrotetracycline necessary to fully induce the P*tetO* promoter in S. gallolyticus subsp. *gallolyticus* UCN34 as 200 ng/ml.

The *blpS* gene was cloned in pTCV-P*tetO* in E. coli and then introduced into WT S. gallolyticus subsp. *gallolyticus* UCN34 and UCN34 Δ*blpS*, and *blpS* expression was induced with 200 ng/ml anhydrotetracycline.

### Transcriptomic analysis and real-time quantitative reverse transcription.

Total RNAs were extracted from exponentially growing S. gallolyticus subsp. *gallolyticus* strains (OD_600_  =  0.5) in THY at 37°C with the MP Biomedicals FastRNA Pro Blue kit following the manufacturer’s recommendations. Bacterial RNA (20 μg) was treated with DNase I (Invitrogen Ambion Turbo DNA-free kit) to remove residual genomic DNA, and then DNase I was inactivated with the recommended reagent.

For whole-transcriptome analysis, rRNA was depleted from 0.5 μg of total RNA using a Ribo-Zero rRNA removal kit (for bacteria) from Illumina. Sequencing libraries were constructed using the TruSeq Stranded mRNA sample preparation kit following the manufacturer’s instructions (no. 20020595; Illumina). The directional libraries were controlled on Bioanalyzer DNA1000 chips (Agilent Technologies), and concentrations were measured with the Qubit double-stranded-DNA (dsDNA) HS assay kit (Thermo Fisher). Sequences of 65 bases were generated on the Illumina HiSeq 2500 sequencer. Reads were cleaned of adapter sequences and low-quality sequences using cutadapt version 1.11 (26). Only sequences at least 25 nucleotides in length were considered for further analysis. Bowtie version 1.2.2 (27), with default parameters, was used for alignment on the reference genome (NC_013798.1 from NCBI). Genes were counted using featureCounts version 1.4.6-p3 (28) from Subreads package (parameters: -t gene -g locus_tag -s 1). Count data were analyzed using R version 3.5.1 and the Bioconductor package DESeq2 version 1.20.0 (29). The normalization and dispersion estimation were performed with DESeq2 using the default parameters and statistical tests for differential expression were performed by applying the independent filtering algorithm. A generalized linear model was set in order to test for the differential expression between the WT, Δ*gsp*, Δ*blpH*, and Δ*blpR* biological conditions. For each pairwise comparison, raw *P* values were adjusted for multiple testing according to the Benjamini-Hochberg procedure (30), and genes with an adjusted *P* value lower than 0.05 were considered differentially expressed.

In the heat maps shown in Fig. 2, gene expression was considered significantly different if the adjusted *P* value was lower than 0.01 and if the log_2_ fold change in gene expression was less than −2 or greater than 2 compared to the value for the WT. Finally, some unassigned genes whose expression was very low (50 to 150 reads per gene) but significantly different in the Δ*blpR* mutant were also suppressed from this heat map for clarity.

For real-time quantitative reverse transcription, cDNAs were obtained from 1 μg of RNA treated with DNase I using the iScript cDNA synthesis kit. Real‐time quantitative PCR was carried out on three independent biological replicates in a CFX96 Touch real-time PCR detection system (Bio-Rad) in a 20-μl mixture containing 10 μl EvaGreen universal qPCR supermix (Bio-Rad), 1 μl gene-specific primers (10 μM), and 5 μl of a 100-fold dilution of cDNA. The fold change in expression compared to WT was determined by the 2^−ΔΔ^*^CT^* method. For statistical analysis, qRT-PCR data were analyzed using analysis of variance (ANOVA): for each gene, a model that explains Δ*C_T_* values was fitted, including the replicate effect as random. The model also includes the strain (Fig. 2C) or the strain, the condition, and their interactions (Fig. 3D) as fixed effects. Pairwise comparisons were tested with the emmeans R package version 1.4.2, and *P* values were adjusted for multiple testing using the Tukey method.

### RACE-PCR.

RACE-PCR to determine the transcriptional start site was performed with the 5′ RACE system (Thermo Fisher) following the manufacturer’s protocol. Briefly, total RNAs were purified from a S. gallolyticus subsp. *gallolyticus* UCN34 WT culture as indicated above. cDNA of *gllA*-*gip* mRNA was obtained by reverse transcription with a gene-specific primer. A homopolymeric tail was added to the 3′ end of the cDNA, corresponding to the former 5′ end of the mRNA. The cDNA was amplified by PCR with another gene-specific primer located in the cDNA and a primer provided in the kit that anneals to the homopolymeric tails of the cDNA. The resulting PCR fragment was cloned in the Zero Blunt TOPO plasmid (Thermo Fisher) and transformed in E. coli. After purification, plasmids were sequenced and sequence alignment was performed to identify the transcription start site.

### Competition.

S. gallolyticus subsp. *gallolyticus* strains were inoculated from fresh agar plates at an initial OD_600_ of 0.05 together with E. faecalis V583 (31) or E. coli *pks*^+^ (32) in the gut microbiota medium and incubated for 5 h at 37°C in a 5% CO_2_ incubator to mimic the anaerobic conditions of the gut. E. faecalis was also inoculated at an initial OD of 0.05 from a fresh agar plate, while E. coli was inoculated at an OD of 0.1 from an overnight culture in gut microbiota medium to overcome its lower growth rate under these conditions. After 5 h of coculture, the mixed cultures were serially diluted and plated on selective agar plates. S. gallolyticus subsp. *gallolyticus* was selected on THY plates containing tetracycline (2 μg/ml), E. faecalis on Entero agar plates, and E. coli on LB plates supplemented with erythromycin (10 μg/ml). CFU were counted the next morning to determine the final concentration (in CFU per milliliter) in each test sample.

### Production and purification of His-tagged recombinant proteins.

Full-length *blpR* and *blpS* were cloned in the pET28a vector in order to obtain 6×His-tagged proteins at their N termini, and after sequence verification, the recombinant plasmids were transferred to the host expression vector E. coli BL21(DE3). Histidine-tagged proteins were purified as previously described (33). Briefly, E. coli cells carrying the plasmid were grown in 500 ml LB supplemented with kanamycin (50 μg/ml) at 37°C with agitation until reaching an OD_600_ of ∼0.5. At this point, 1 mM IPTG (isopropyl-β-d-thiogalactopyranoside) was added to the culture to induce protein expression, and the culture was incubated for 3 h at 37°C with agitation. Bacteria were pelleted by centrifugation (5,000 × *g* for 10 min) and resuspended in 20 ml of lysis buffer (33) containing 1 mg/ml of lysozyme. Cell debris was eliminated by centrifugation (9,000 × *g* for 30 min), and 1 ml of Ni-NTA Superflow beads (Qiagen) was added to bind His-tagged proteins. After being wash on a gravity flow column, His-tagged proteins were eluted with an elution buffer containing 500 mM imidazole (33). Fractions containing the recombinant protein were pooled and resuspended in the buffer (50 mM NaH_2_PO_4_, 300 mM NaCl, 1 mM dithiothreitol [DTT], 20% glycerol; pH 8) using PD10 columns. Purified proteins were conserved at −80°C. Just before use, proteins were concentrated around 10-fold on Vivaspin column (5-kDa cutoff), and protein concentration was estimated with a Nanodrop instrument by OD_280_ measurement.

### DNA-protein interactions.

Electrophoretic mobility shift assay (EMSA) and footprinting were performed as described previously (34). Briefly, promoter sequences (Table 3) of about 150 bp were amplified by PCR and cloned in the pTCV-lac vector (35). The *gllA* promoter and its scrambled derivative were synthesized by Genecust and cloned in the pTCV-lac vector. All promoters were then amplified by PCR with radiolabeled primers specific for plasmid cloning site (VlacE and VlacB). Radiolabeled PCR fragments were diluted 100-fold and incubated for 20 min in binding buffer (25 mM Na_2_HPO_4_/NaH_2_PO_4_ [pH 8], 50 mM NaCl, 2 mM MgCl_2_, 1 mM DTT, 10% glycerol) supplemented with 0.02 μg/μl bovine serum albumin (BSA) and 0.1 μg/μl of poly(dI-dC) (Sigma) in the presence of serial 2-fold dilutions of purified BlpR/BlpS or buffer. After migration of the different reaction products on a 6% polyacrylamide gel for 1 h, gels were analyzed by autoradiography.

**TABLE 3:** Sequences of the promoters tested by EMSA

Promoter	Sequence^*a*^	Length (bp)
P*gllA*	ACGGTCAAAAAATCATGAAAATACAAAAAAATTTACCATTT**ACGACCTAAAAACAACAAATTACGACTTGGG** CGTGCATTAACCTCACAATCTGTTAGACTAAAATCTGTCAGTTGCAACGACAAAATATTAT	133
P*gllA*-scr	ACGGTCAAAAAATCATGAAAATACAAAAAAATTTACCATTTA**AGTAAGATCAGTACAATAACCTACACAGGC** CGTGCATTAACCTCACAATCTGTTAGACTAAAATCTGTCAGTTGCAACGACAAAATATTAT	133
P*gsp*	GATGGCTTGGACTTTTTCAATATTCTTTGTTGCCGTTTACGACCGAAAAATGACTTTTTATGACTTAGAAAAA GCTTTTAAGAACTTTTCTGCTACAATTAATCACTAAGGAGGTTTATAAAAATGA	127
P*blpA*	TTTGGAAGAATGGTAAAGAAAAATAATTTTTTGACCGTTTGCGACCTATTTTTGACTTTTTACGACTTGGGCG TTTTTTAATCTTGTTTTATGGCACAATAGTTCTAGTTTCATTTTTAAAAGGGAAAGGTTATGAGAAG	140
P*gyrA*	TAAGTGAGATATGTCACGAACTAAATTGTAAAAACTTTGAAAAAAAGAAGAAAGCTAGTGATTCTGGCGAAAAA GCGCTATTTTTGCCAAAAATGTGGTATAATATAGTAGAGTTTTACACTAGAAAAGGAGTGTTTTTCCTTA	144

a The P*gllA* sequence that is scrambled in P*gllA*-scr is in bold.

The same binding conditions were used for footprint experiments. After binding, DNA was digested with 62.5 ng/ml of DNase I (Worthington Biochemical) for 30 s at room temperature. The reaction was then stopped, and the DNA was purified by phenol extraction and ethanol precipitation. Purified DNA was migrated on 6% polyacrylamide–7 M urea sequencing gels that were analyzed by autoradiography. Maxam-Gilbert reactions (A+G) were carried out on the same promoters to determine their sequence and precisely determine the region protected by the binding of the regulator.

### BlpR phosphorylation.

*In vitro* phosphorylation was performed by incubating BlpR at 37°C for 1 h in the presence of 35 mM acetyl phosphate (Sigma) and 20 mM MgCl_2_.

### Data availability.

The raw data for the transcriptomic analysis are available on GEO data server (accession number GSE148401).
