# Supplementary figures and images for: Characterization of a Four-Component Regulatory System Controlling Bacteriocin Production in Streptococcus gallolyticus
Source: mBio. 2021 Jan 5;12(1):e03187-20. doi: 10.1128/mBio.03187-20 (PMC8545106; doi:10.1128/mBio.03187-20)

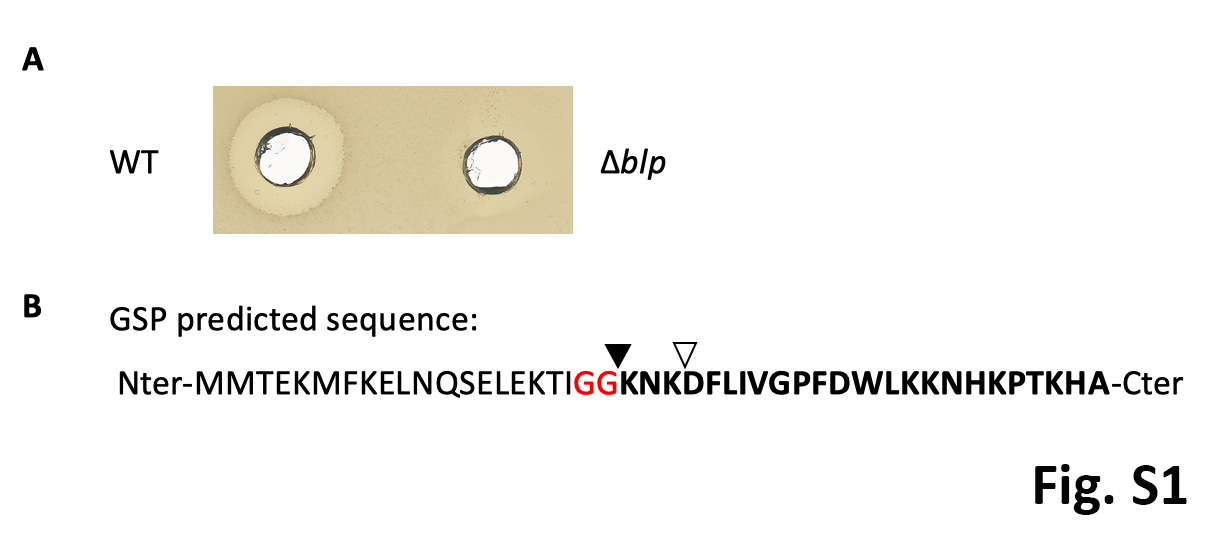

Supplement: FIG S1 [file mbio.03187-20-sf001.tif]

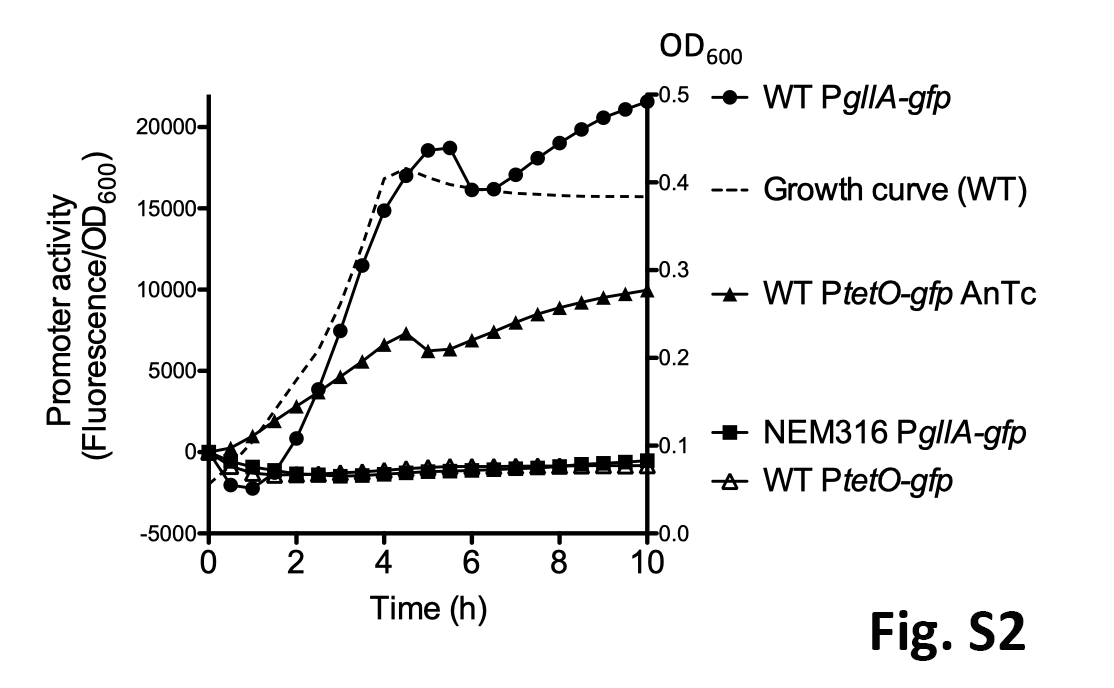

Supplement: FIG S2 [file mbio.03187-20-sf002.tif]

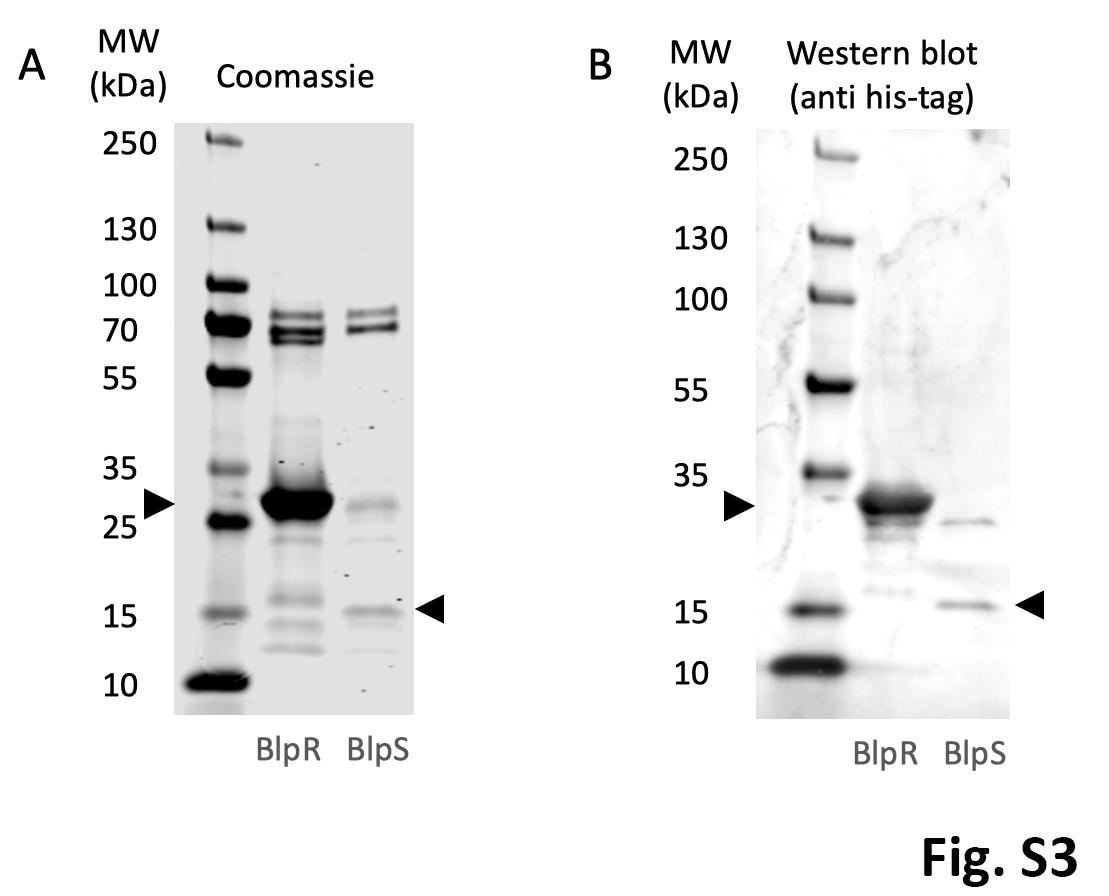

Supplement: FIG S3 [file mbio.03187-20-sf003.tif]

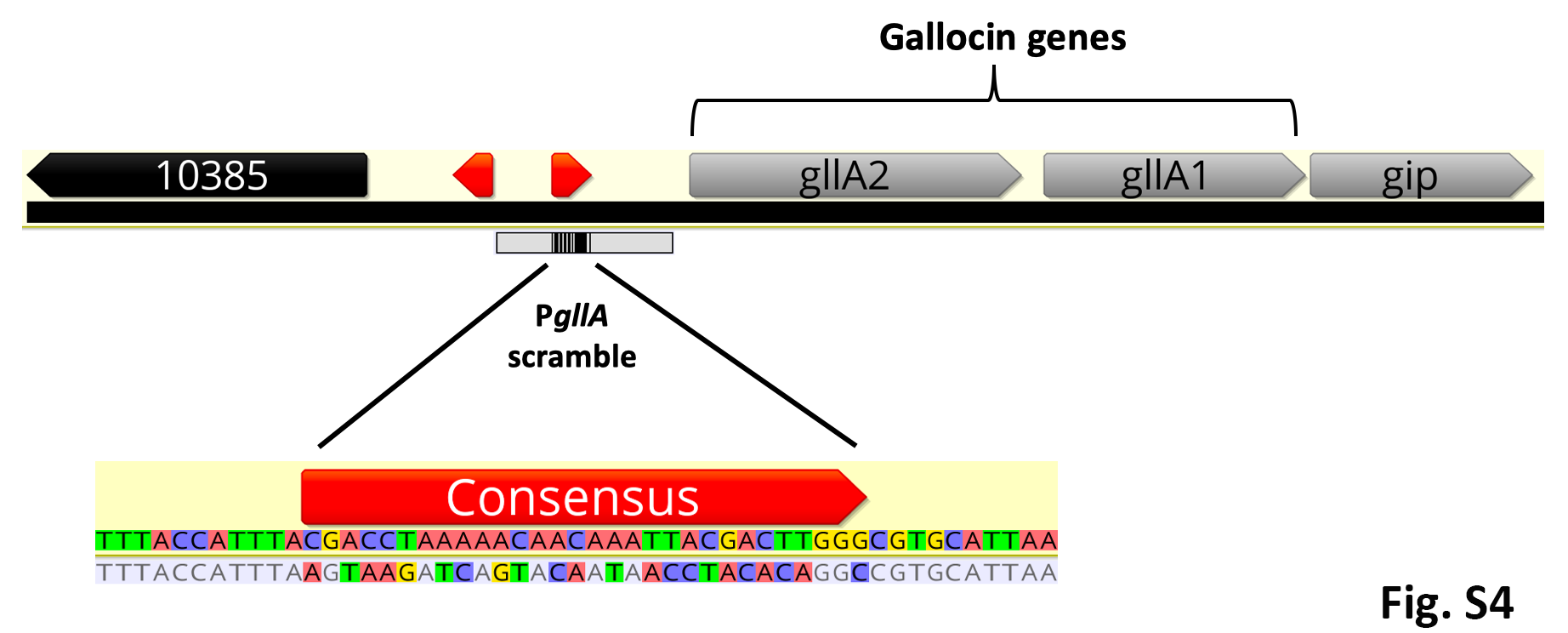

Supplement: FIG S4 [file mbio.03187-20-sf004.tif]

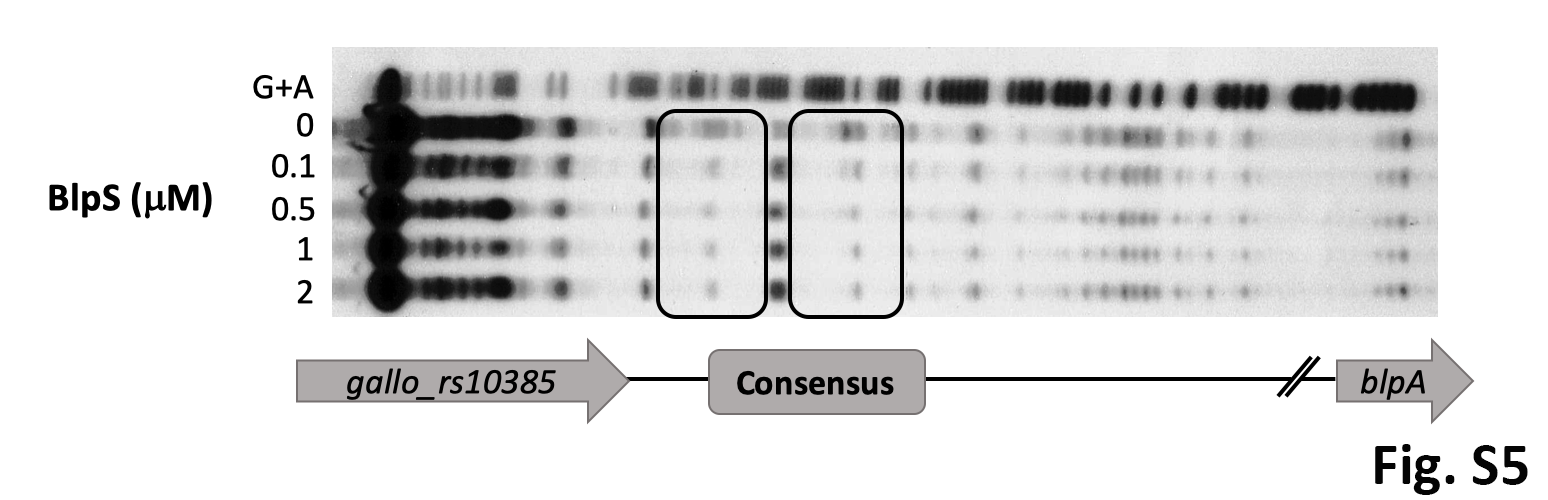

Supplement: FIG S5 [file mbio.03187-20-sf005.tif]

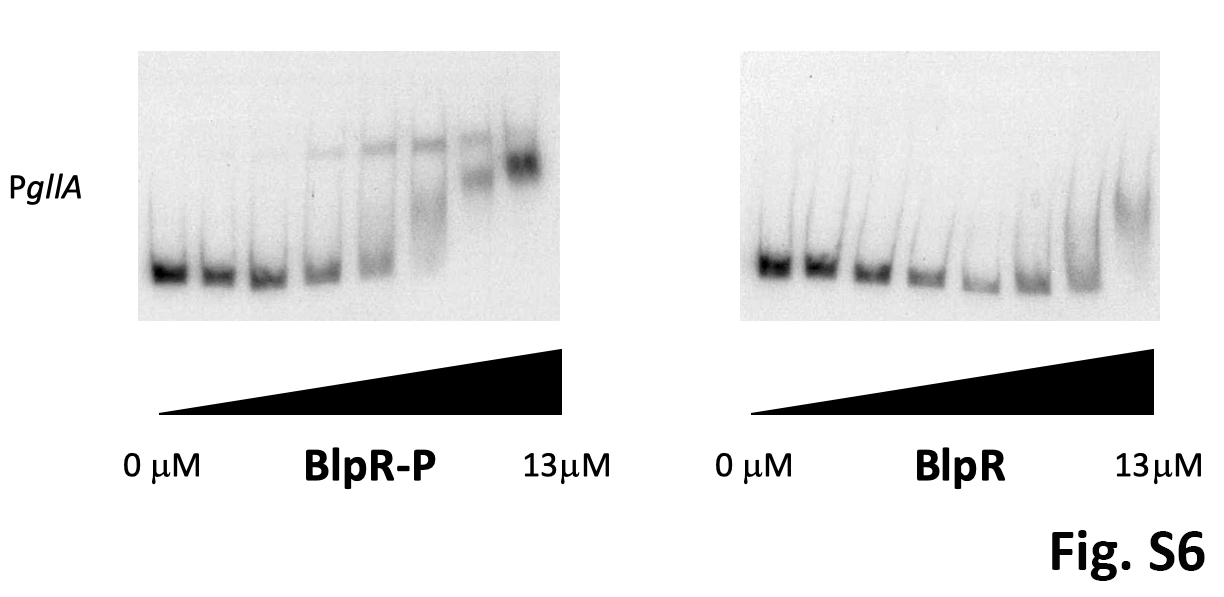

Supplement: FIG S6 [file mbio.03187-20-sf006.tif]

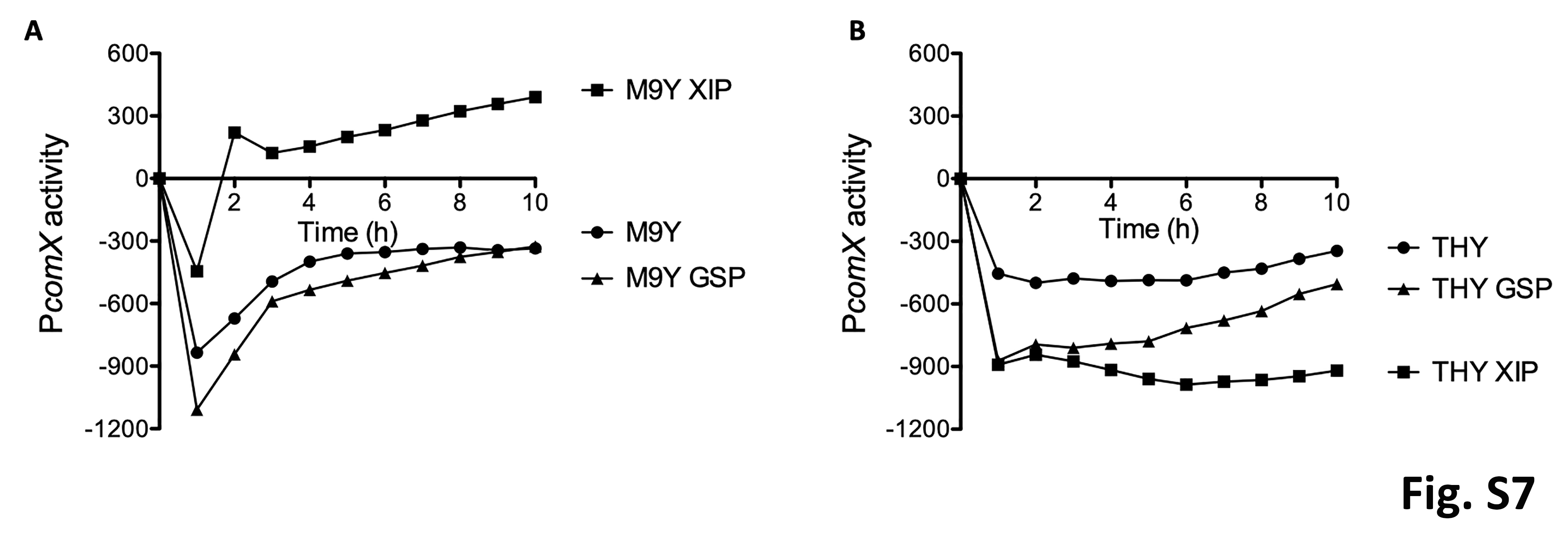

Supplement: FIG S7 [file mbio.03187-20-sf007.tif]
